# Kinetically Stable Boron‐Heteroatom Bonds

**DOI:** 10.1002/anie.6307397

**Published:** 2026-07-30

**Authors:** Alina Trofimova, Ben Zhen Huang, Harjeet S. Soor, Brandon White, Aleksandra Holownia, Yury Lebedev, Alan J. Lough, Travis Dudding, Andrei K. Yudin

**Affiliations:** ^1^ Davenport Research Laboratories Department of Chemistry University of Toronto Toronto Ontario Canada; ^2^ Department of Chemistry Brock University St. Catharines Ontario Canada

**Keywords:** boryl azide, boryl halide, hemilability, kinetic stability

## Abstract

Achieving kinetic stability of hydrolytically labile bonds remains a persistent goal of chemistry. The present study investigates the kinetic stability of boron‐heteroatom bonds in *N*‐methyliminodiacetic acid (MIDA) boronates. The hemilabile nature of the ligand has enabled the synthesis of molecules that would otherwise be considered too hydrolytically labile. Depending on the structure, acid‐promoted migration of the boryl substituent was found to produce molecules with boron bound to nitrogen in different oxidation states. The formation of azidoboronate over aminoboronate is a particularly interesting consequence of kinetic stabilization and is at odds with the expectedly low‐energy path to nitrogen gas elimination. By focusing on the unusual stability of boron‐heteroatom bonds in different settings, this study contributes to the development of fundamental aspects of organoboron chemistry and offers a path to versatile building blocks that should find widespread utility in chemistry.

## Graphical abstract

This study investigates the kinetic stability of boron‐heteroatom bonds in *N*‐methyliminodiacetic acid (MIDA) boronates. The hemilabile nature of the ligand enables the synthesis of molecules that would otherwise be considered too hydrolytically labile. Depending on the structure, acid‐promoted migration of the boryl group was found to produce compounds with boron bound to nitrogen in different oxidation states. The research contributes to the development of fundamental aspects of organoboron chemistry.

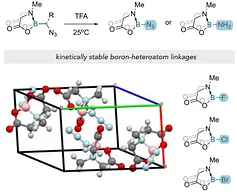

## 1 Introduction

Achieving kinetic stability of hydrolytically labile bonds is an enduring objective of chemistry. Several factors, such as steric hindrance [1, 2], conformational rigidity [3, 4], and non‐covalent interactions [5], can enhance the kinetic stability of labile linkages. Dative bonds can play pivotal roles in fortifying comparatively fragile connections [6]. This paper focuses on boron‐heteroatom bonds, which generally exhibit low kinetic and thermodynamic stability in polar reactions. For instance, boron halides are known to react vigorously with water to yield boric acid and the corresponding hydrogen halide [7, 8, 9]. Monomeric B─O bond‐containing molecules succumb to oligomerization, whereas boryl azides rearrange photolytically or thermally to iminoboranes with the release of dinitrogen [10, 11, 12, 13, 14, 15, 16, 17, 18]. We have been intrigued by the factors that govern kinetic stabilization of boron‐heteroatom bonds and now report our findings in this area.

**FIGURE 1:**
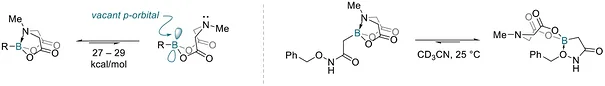
Amine hemilability in MIDA boronates.

Past studies have established *N*‐methyliminodiacetic acid (MIDA) [19] as a powerful ligand that enabled the isolation of a range of previously inaccessible boron‐containing species [20, 21, 22, 23, 24]. The hemilabile nature of the B─N bond in the MIDA ligand allows boron to behave as either electrophile or nucleophile [24]. While the barrier to nitrogen decoordination in MIDA boronates of ∼27 kcal/mol suggests a fairly slow process with a half‐life on the order of 100 days [25], proximity of boron to heteroatoms can lower the barrier and enable hemilability (Figure 1) [24, 26]. Amine decoordination previously allowed us to trap electrophilic palladium complexes generated under C─H activation conditions [27]. In marked contrast to these findings, we now report the unusual kinetic stability of boron‐heteroatom bonds. Our study provides insights into the fundamental aspects of bonding, challenges long‐held assumptions about inorganic azides, and describes unusually stable boron‐containing azides, boryl triazines, as well as the first example of a fundamentally important monomeric B─NH_2_ functionality that can be selectively modified at nitrogen.

## 2 Results and Discussion

**FIGURE 2:**
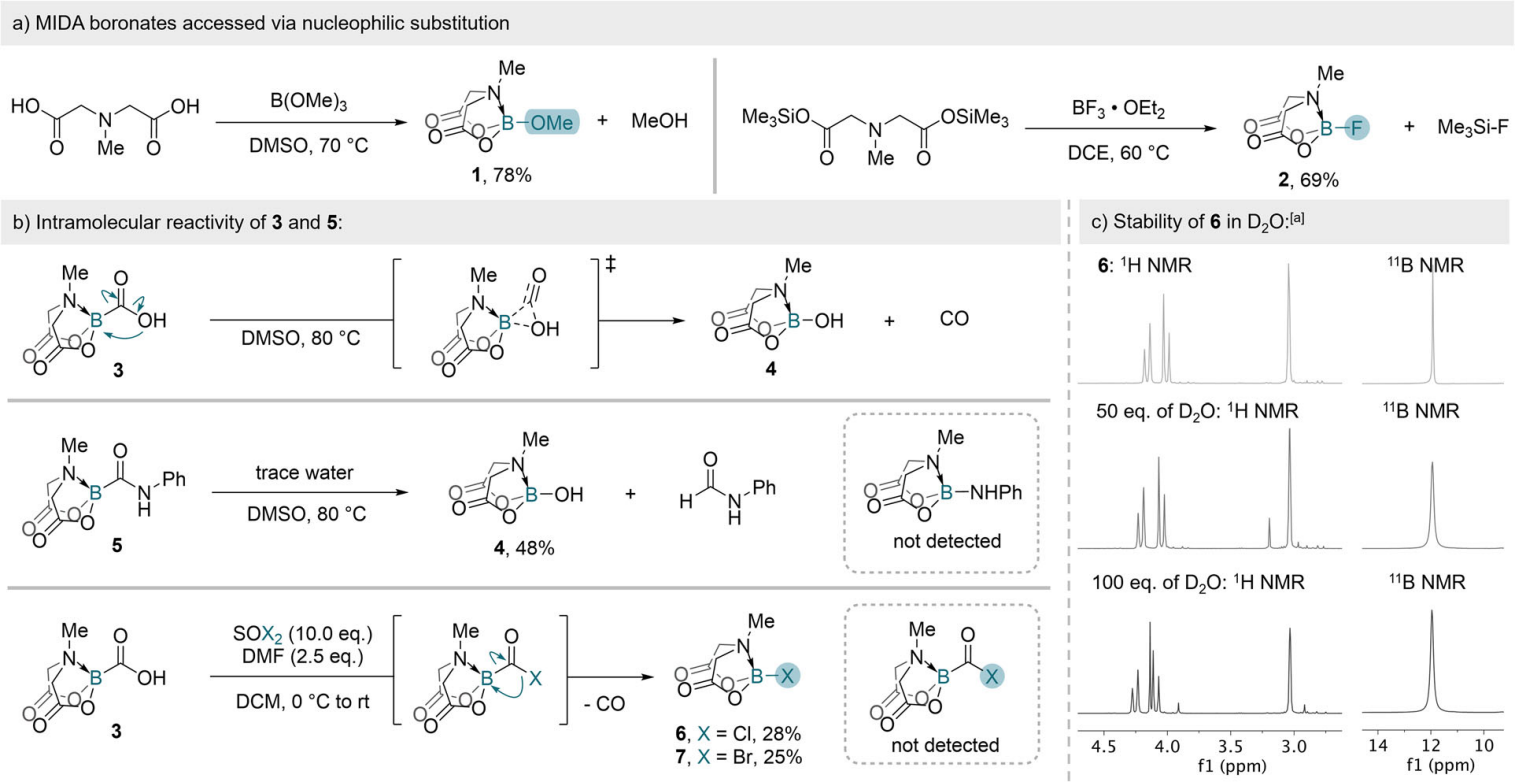
(a) Synthesis of methoxyboronate **1** and fluoroboronate **2**; (b) synthesis of hydroxyboronate **4**, chloroboronate **6,** and bromoboronate **7**; and (c) stability of **6** in D_2_O over 5 days. ^a^Measured in CD_3_CN at room temperature.

The hemilability of the B─N bond in MIDA boronates allows boron to behave as either electrophile or nucleophile [24] Consequently, we surmised that novel boron‐heteroatom linkages could be accessed by nucleophilic substitution at boron. Methoxyboronate **1** and fluoroboronate **2** were synthesized by ligand exchange using commercially available B(OMe)_3_ and BF_3_•OEt_2_ as boron sources and MIDA and TMS_2_MIDA as nucleophiles, respectively (Figure 2a). These reactions required no column chromatography, as MIDA boronates could be purified by washing the crude mixture with diethyl ether. NMR analysis revealed a distinct AB‐quartet [28] in ^1^H NMR spectra corresponding to the diastereotopic methylene protons of the MIDA backbone and ^11^B NMR chemical shifts of 9.1 and 8.2 ppm for **1** and **2**, respectively. Previously, MIDA‐containing thiophene, selenophene, and tellurophene rod‐like molecules were shown to self‐assemble into permanently porous hydrogen‐bonded organic frameworks (HOFs) through multiple hydrogen bonding interactions [29]. Because of the possibility to vary the substituent on oxygen, our novel alkoxyboronates may also find applications in materials science. Upon subjecting **1** or **2** to external nucleophiles such as sodium azide in DMF, however, only the starting material was recovered. In contrast to our previous reports where MIDA hemilability was demonstrated in the presence of nucleophiles [24], the reaction conditions did not promote B─N decoordination in the present case. The relative inertness of **1** and **2** was further evidenced in reactions with TMSOTf or hydride delivery agents such as NaBHEt_3_ and LiAlH(*t*BuO)_3_ (see Supporting Information).

As MIDA boronates **1** and **2** were not prone to nucleophilic substitution, we turned our attention to rearrangements. Previous studies by our group revealed that carboxy‐MIDA boronate **3** releases CO gas under neutral reaction conditions [30]. Upon thermal activation, compound **3** undergoes decarbonylation through a cyclic three‐membered transition state (Figure 2b) [30, 31, 32, 33], in which cheletropic fragmentation affords hydroxyboronate **4** and CO. Based on these results, we decided to investigate if other nucleophiles could participate in the rearrangement. When carbamoyl‐MIDA boronate **5** was heated to 80°C, hydroxyboronate **4** was isolated in 48% yield in addition to formanilide as the by‐product. The anticipated aminoboronate was not detected in the reaction mixture. Compound **4** could be generated either via protodeboronation [34] of **5** or through a three‐membered transition state similar to our previously reported case of carboxyboronate. The boric acid derivative **4** was also generated when thiocarbo‐MIDA boronate was heated to 80°C in DMSO (see Supporting Information).

Building on a collection of borylated compounds, we anticipated that acid chloride could be obtained upon subjecting **3** to SOCl_2_ in DMF. The expected product was not detected by NMR analysis. Instead, an unknown material with a ^11^B NMR chemical shift of 11.9 ppm was generated. This compound was stable to purification by normal‐phase column chromatography. Upon characterization by NMR spectroscopy and HRMS, its identity was confirmed to be that of chloroboronate **6** (Figure 2b). We found that **6** could also be obtained by reacting BCl_3_ with TMS_2_MIDA through nucleophilic substitution. Based on the identification of **6**, we propose that the reaction goes through a three‐membered transition state similar to the carboxyboronate case. Upon formation of the acid chloride intermediate, attack of the chloride at the boron center forms the B─Cl bond while concomitant cleavage of the B─C bond affords chloroboronate **6** with the release of CO. Analogously, bromoboronate **7** was synthesized from **3**. Tricoordinate boron‐halogen bonds are known to be labile, producing HCl or HBr upon treatment with water [7, 8, 9]. Tetracoordinate boron complexes with boron‐halogen bonds also hydrolyze over a few hours of exposure to water at room temperature [35]. To our surprise, no reaction was observed when **6** was subjected to 50 or 200 equivalents of D_2_O for several days, highlighting the unusual kinetic stability of the new species (Figure 2c). In contrast, methoxyboronate **1** decomposed to boric acid and methanol upon subjection to D_2_O overnight (see Supporting Information). Thus, unlike previously known aryl MIDA boronates, **6** did not undergo hydrolysis [25] under neutral conditions. Base‐mediated hydrolysis of MIDA boronates generally proceeds within 10 min [25]. When subjected to aqueous NaOH solution, **6** did not fully convert to boric acid even after prolonged reaction times (see Supporting Information).

**FIGURE 3:**
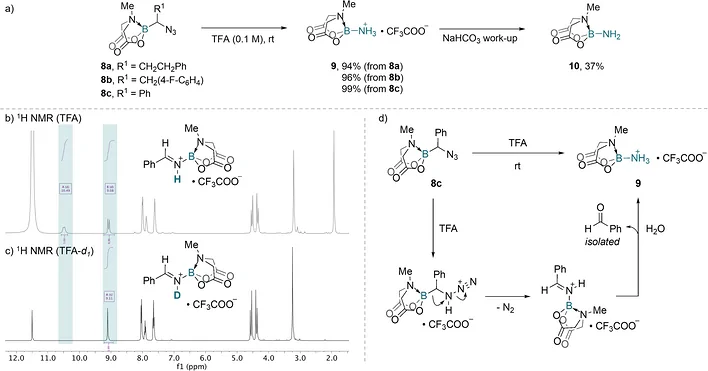
(a) Synthesis of aminoboronate **10** and its trifluoroacetate salt **9**; (b) ^1^H NMR spectrum of the reaction between **8c** and TFA; (c) ^1^H NMR spectrum of the crude reaction between **8c** and TFA‐*d_1_*; (d) proposed mechanism for the formation of **9** from **8c**; and (e) N‐acylation of **10** and the X‐ray structure of **11**.

As aminoboronate was not afforded through the rearrangement of **5**, we wanted to access this molecule by other means. Since the BMIDA group has shown to exhibit high migratory aptitude in polar rearrangements [24, 36, 37], we envisioned that the azido‐Schmidt reaction of boryl azides could yield the desired amine [38]. When α‐substituted azides **8a**, **8b**, and **8c** were reacted with TFA, aminoboronate trifluoroacetate salts **9** were isolated in nearly quantitative yields (Figure 3a). The corresponding free aminoboronate **10** was afforded after basic workup. Considering the growing interest in nanotube‐boramidic acid derivatives for sensing applications, this simple building block can find applications in materials science because it offers the first instance of a monomeric building block with B─NH_2_ linkage that is nucleophilic at nitrogen [39]. The in situ reaction monitoring through NMR analysis revealed the formation of *N*‐BMIDA‐iminium ion species in all three cases. For instance, in the case of **8c**, peaks at 9.08 and 10.49 ppm (*J *= 19.0 Hz, corresponding to the *trans*‐geometry of the iminium protons) in ^1^H NMR spectrum appeared when the reaction was conducted in non‐deuterated TFA (Figure 3b) [38, 40]. The disappearance of the splitting pattern was observed when TFA‐*d_1_* was employed, as the deuterium replaced the proton on the nitrogen atom (Figure 3c). Benzaldehyde was detected in situ and isolated as the only by‐product of the reaction. A reasonable reaction mechanism is shown in Figure 3d. In the presence of acid, boryl‐alkyl azide **8** gets protonated. The boryl group migrates to the nitrogen atom with the release of dinitrogen, which is consistent with the experimentally observed gas evolution, to afford the boryl iminium ion. Recently, we have shown that the boryl group in acyl‐MIDA boronates migrates in preference to the alkyl group in the Roskamp rearrangement, releasing N_2_ gas [36]. In the present case, the BMIDA group also migrates in preference to the phenyl group. The generated boryl iminium ion is stable in solution but undergoes hydrolysis upon aqueous workup to yield aminoboronate trifluoroacetate salt **9** and benzaldehyde.

**FIGURE 4:**
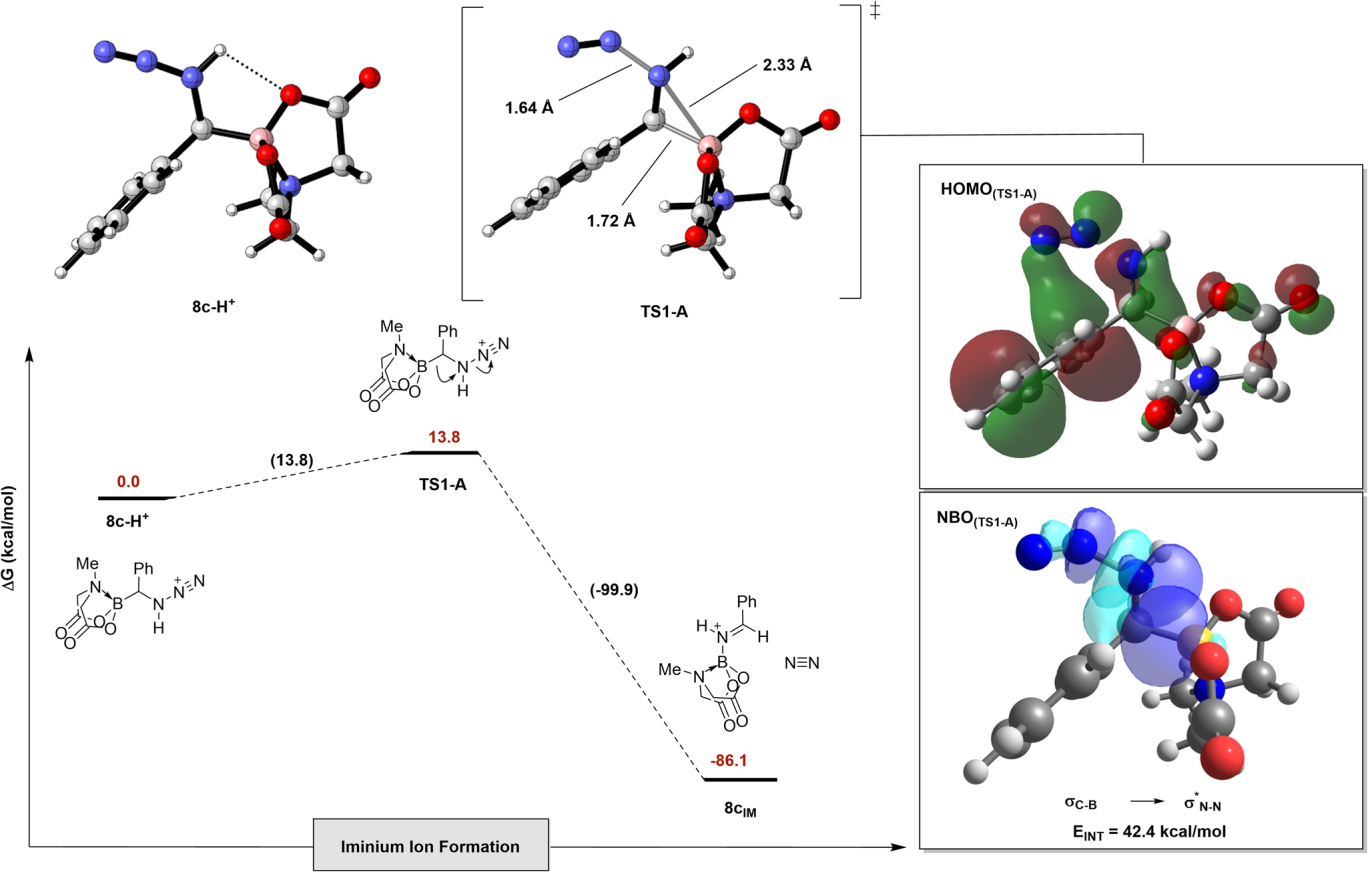
DFT calculated pathway for iminium ion formation. All structures were computed at the IEFPCM_(TFA)_ωB97X‐D/6‐31G+(d,p) level of theory. The figure includes HOMO and NBO interaction of **TS1‐A**.

To further highlight the applications of aminoboronate **10**, it was subjected to a reaction with acyl chloride (Figure 3e). Compound **10** displayed typical amine reactivity producing borylated benzamide **11**, the structure of which was confirmed by X‐ray crystallography. To gain further insight into the mechanism of the formation of aminoboronate trifluoroacetate salt **9**, density function theory calculations were performed at the IEFPCM_(TFA)_ωB97X‐D/6‐31G+(d,p) level of theory (Figure 4) [41, 42, 43]. Our investigation commenced with an examination of the intrinsic reactivity of protonated boryl alkyl azide **8c‐H^+^**, featuring an intramolecular N─H···O hydrogen bond between the azide and BMIDA ester oxygen. This interaction locks the B─C *σ*‐bond in an *anti*‐periplanar orientation relative to the azide group, thereby preorganizing the intermediate for dinitrogen extrusion via **TS1‐A**. The computed Gibbs free energy of activation (Δ*G* ^‡^) for this transformation is 13.8 kcal/mol, consistent with a kinetically accessible process under mild conditions. The defining bond‐breaking distances of this boryl migration structure include elongated N─N and C─B bonds measuring 1.64 and 1.72 Å, respectively, concurrent with incipient B─N bond formation at 2.33 Å. Further natural bond orbital (NBO) analysis revealed a pronounced donor–acceptor interaction between the B─C *σ*‐bond and the breaking N─N anti‐bond (*σ*_C‐B_ → σ*_N‐N_ orbital, *E*_INT_ = 42.4 kcal/mol) contributing to bond cleavage linked to a hydridic type migration of BMIDA. Consistent with this picture, the **TS1‐A** exhibits in‐phase orbital density localized along the B─C─N linkage with an out‐of‐phase component localized on the cleaving N─N bond, illustrating the underlying orbital interactions contributing to concerted boryl group migration and dinitrogen extrusion. From **TS1‐A**, formation of the ensuing iminium ion intermediate (**8c_IM_**) is predicted to be highly exergonic (–99.9 kcal/mol), providing a strong thermodynamic driving force for the overall process.

**FIGURE 5:**
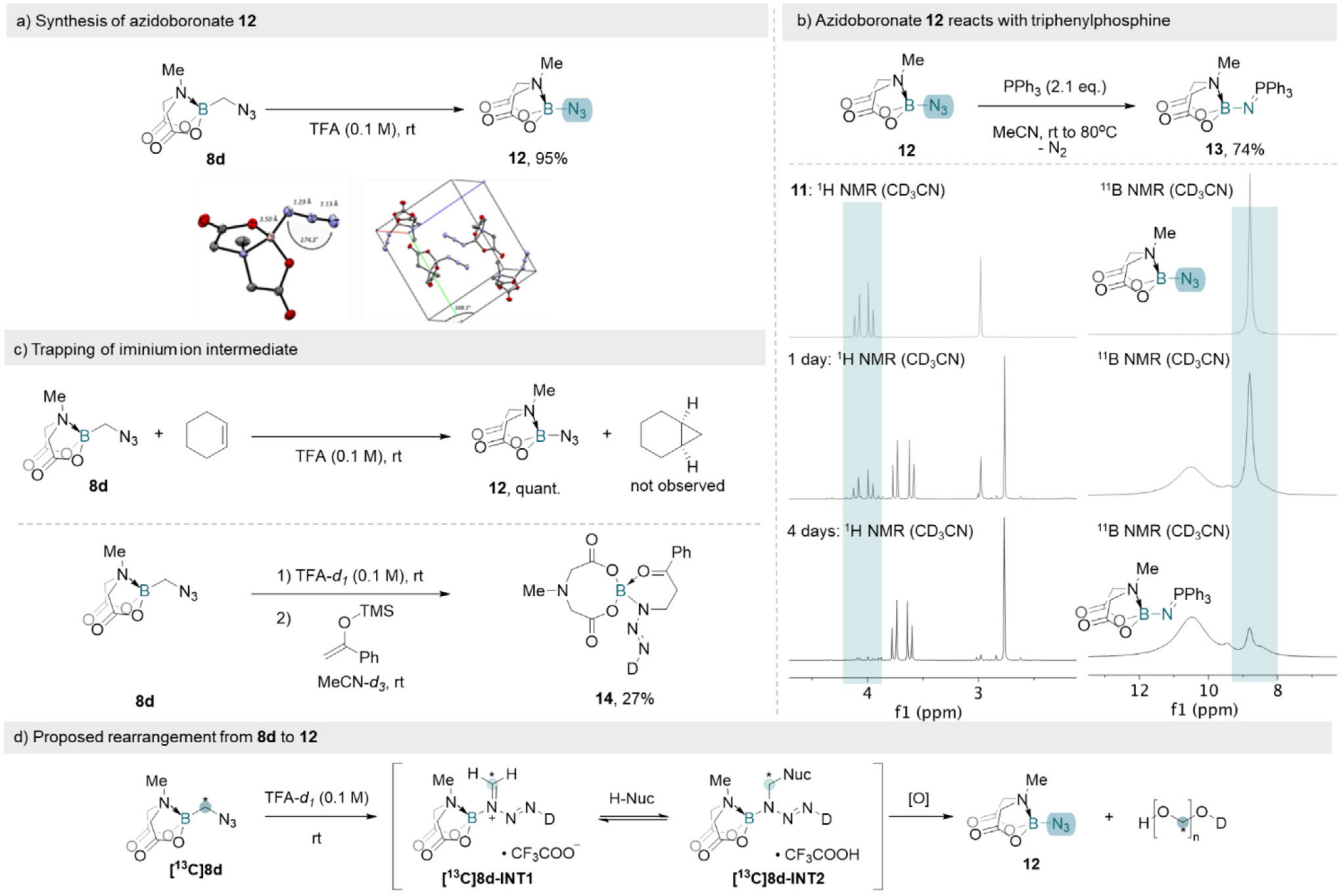
(a) Synthesis of azidoboronate **12** and its X‐ray structure. Hydrogens are omitted for clarity. B: pink, N: blue, O: red, C: gray. (b) Synthesis of **13**: ^1^H and ^11^B NMR (in CD_3_CN) data showing conversion of starting azidoboronate **12** to iminophosphorane **13**. (c) Attempted trapping of the carbene intermediate with cyclohexene and phenylvinyl trimethylsilyl ether. (d) ^13^C‐labeled mechanistic studies.

Since all three boryl‐alkyl azides (**8a**‐**8c**) produced aminoboronate **10**, we opted to examine how the lack of substitution at the α‐position could influence reactivity. We subjected unsubstituted boryl‐alkyl azide **8d** [44] to TFA at room temperature and observed the formation of a new boron‐containing species – azidoboronate **12** (Figure 5a). Boron‐bound azides rearrange photolytically or thermally to iminoboranes with the release of dinitrogen [18], which makes the observed stability rather remarkable. The product was amenable to aqueous workup and column chromatography. The ^11^B NMR resonance shifted from 10.8 ppm to 8.7 ppm. The incorporation of an azide group was also evident from the IR spectrum (strong band at 2160 cm^−1^, see Supporting Information) [45]. Further confirmation of the product was achieved through X‐ray crystallography. Analysis of the crystal structure showed monoclinic packing, characteristic of azides bound to small metals. The B–N_α_ bond length of 1.50 Å of **12** is typical of boron‐azide bonds [46]. The azide angle present in our system was found to be 174.3°, showing slight out‐of‐plane bending of the azide moiety. The N_α_–N_ß_ and N_ß_–N_γ_ bond lengths in the azide are 1.23 Å and 1.13 Å, respectively, indicating a large triple bond character between N_ß_ and N_γ_.

We have found that the N_ß_–N_γ_ bond of **12** was shorter than that of typical azides reported in the literature [46]. Studies of inorganic azides performed by Wilkinson and Cartwright have shown a positive correlation between a bond length and sensitivity to explosions based on a) decomposition induced by simple electronic rearrangements and b) little atomic movement [47]. With a carbon‐to‐nitrogen ratio of 2.25, one can imagine **12** to be explosive. Highly reactive species composed of boron and nitrogen atoms have been investigated by the Braunschweig group [48]. Curiously, we did not observe any explosive behavior while handling our azide. Thermogravimetric analysis (TGA) of **12** indicated the loss of mass corresponding to dinitrogen only above 200^°^C. In addition, the melting of **12** (m.p.: 174^°^C–175^°^C) occurred with no gas formation, indicating true melting properties.

Having successfully synthesized **12**, we wondered if this azidoboronate would react as an azide delivery agent similar to TMSN_3_, which is a safer analog of HN_3_ [49]. The reactions involving the azide moiety, such as cycloadditions, substitutions, and ring expansions did not occur. Compound **12** showed lack of reactivity in the presence of TBAF and H_2_O, which was surprising given the relative strengths of B─F and B─O bonds. We hypothesized that the N_α_ atom on azidoboronate has reduced nucleophilicity due to the steric hindrance from the MIDA moiety and reduced electron density through a donation from the azide nitrogen to boron. The Bettinger group has previously shown that catechol azidoboronates decompose photochemically to the corresponding boryl nitrenes [15, 16, 17]. To our surprise, no reaction was observed when **12** was irradiated with blue light and an iridium catalyst (or in its absence) for 3 days, and only the starting material was recovered. Azides are also known to react with rhodium complexes to produce metal nitrenes, reactive intermediates used in C─H insertion and alkene aziridination [50]. When **12** was subjected to nitrene‐forming conditions, no conversion was observed even at elevated temperature.

Exploring the reactivity of azidoboronate, we found that exposure of **12** to triphenylphosphine resulted in no iminophosphorane formation at room temperature. This lack of reactivity contrasts with those reported in the literature by Stephan and coworkers where the low barriers of iminophosphorane formation were attributed to the use of an electron‐rich phosphine but also to the electron‐withdrawing capabilities of the *sp^2^* boron center [51]. Iminophosphorane **13** was formed only at elevated temperatures, as confirmed by ^1^H, ^11^B, and ^31^P NMR analyses (Figure 5b). Upon heating to 80°C overnight, a new set of diastereotopic MIDA methylene protons appeared in the ^1^H NMR spectrum. The ^11^B NMR showed a peak at 10.5 ppm, downfield of the peak of the starting material (8.8 ppm), which was assigned to **13**. Even after prolonged reaction time, full conversion of **12** was not observed. Captain and Hoff have demonstrated that the reaction of equimolar amounts of PR_3_ (*R* = Cy or *i*Pr) with bulky 1‐adamantylazide (N_3_Ad) in aromatic solvents at room temperature results in the equilibrium with the R_3_PN_3_Ad adduct [52]. The bulky phosphazide adducts R_3_PN_3_Ad are kinetically stable at ambient temperatures in the absence of light to N_2_ loss. Despite similar size, the adducts of **12** and PPh_3_ were not observed in our case. The isolated **13** is a rare example of iminophosphorane with boron in a tetracoordinate form [53, 54]. As bifunctional iminophosphorane catalysts have gained interest, for example, in enantioselective desymmetrization of phosphorus compounds [55, 56, 57], **13** and its chiral derivatives can potentially find applications in catalysis. In addition, azidoboronate **12** is a likely candidate for accessing borylated heterocycles [51, 58].

The formation of azidoboronate **12** from **8d** was unexpected based on the known behavior of the azide moiety. The loss of a methylene fragment over nitrogen gas expulsion is unprecedented. The possibility of gas evolution in the reaction course was ruled out, as only TFA vapors were detected by the headspace analysis by GC‐TCD. A control reaction conducted with silver trifluoroacetate instead of TFA indicated that the presence of an acidic proton was necessary for the reaction to occur. Intrigued by the possibility of carbene generation, we attempted to trap it in the reaction with cyclohexene under standard conditions (Figure 5c). Yet, none of the expected products were observed. We then attempted to trap a possible iminium ion intermediate with a silyl enol ether (Figure 5c). Triazine **14** was isolated in the reaction with phenylvinyl trimethylsilyl ether, indicating that the reaction had proceeded through the generation of a boryl iminium ion. Retention of the partially reduced azide moiety in triazine **14** was confirmed by HRMS. The molecule was found to be stable to aqueous workup and column chromatography. To the best of our knowledge, triazine **14** is the first report of a partially reduced organic azide. Notably, ^1^H NMR spectrum of **14** showed enantiotopic MIDA methylene protons, suggesting displacement of the coordinated amine by ketone oxygen in the product (see Supporting Information).

To gain additional mechanistic evidence, the conversion of **8d** to azidoboronate **12** was monitored by NMR spectroscopy. Two doublets at 8.39 and 8.30 ppm appeared in the ^1^H NMR spectrum when the reaction was conducted with deuterated TFA (see Supporting Information). The observed peaks were similar to those of methanimine triethylborane complexes previously reported by the Brown group [40] and *N*‐phenyl iminium ions reported by the Aube group [38]. The HSQC NMR experiment of the crude reaction mixture revealed the cross‐peak of the vinyl protons and the carbon atom with a chemical shift of 168.0 ppm. Furthermore, we synthesized ^13^C‐labeled azidoboronate **[^13^C]8d** and subjected it to TFA‐*d_1_* (Figure 5d). The splitting of iminium ion protons by ^13^C‐nuclei was observed in the ^1^H NMR spectrum (*J* = 187.0 Hz) of the crude reaction mixture. The peak at 87.3 ppm was also detected in the ^13^C NMR spectrum, indicating the formation of polymerized formaldehyde (H[O^13^CH_2_]_n_OD) (see Supporting Information). Based on the experimental results and previously reported literature, we hypothesize that upon the addition of acid, protonation of the terminal nitrogen of azide takes place, followed by 1,2‐boryl migration affording the iminium ion intermediate detected by ^1^H NMR and the ^13^C‐labeled studies. The driving force of the transformation is the formation of a strong B─N bond as opposed to a B─C bond in the starting material, which acts as a thermodynamic sink to bypass the nitrogen expulsion pathway. The avoidance of the Schmidt pathway was previously seen by the Ghorai group in azidocarbenium species trapping [59]. After that, nitrogen–carbon bond cleavage takes place, producing azidoboronate **12** and polymerized formaldehyde.

**FIGURE 6:**
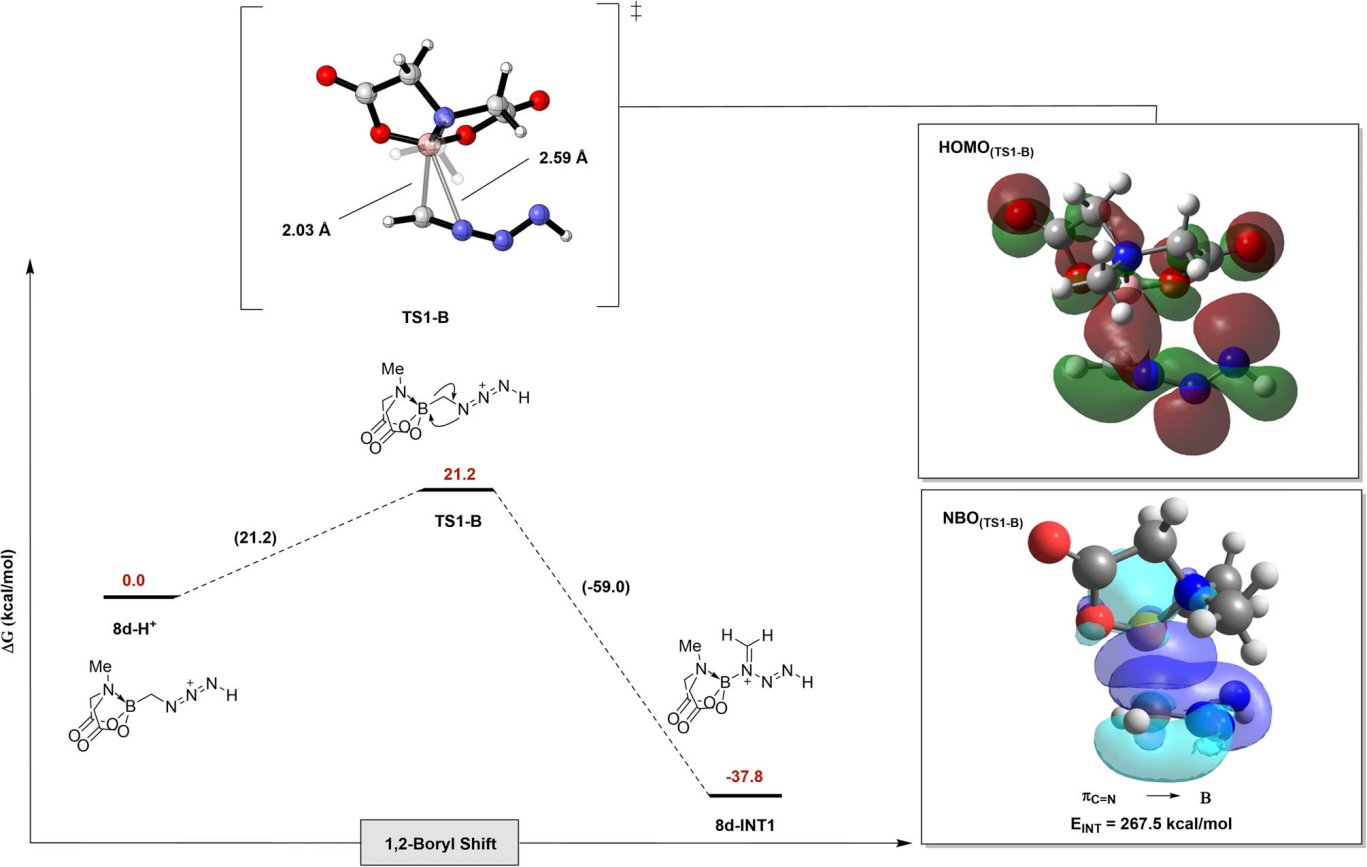
DFT calculated pathway for rearrangement and iminium ion formation **8d‐INT1**. All structures were computed at the IEFPCM_(TFA)_ωB97X‐D/6‐31G+(d,p) level of theory. The figure includes HOMO and NBO interaction of **TS1‐B**.

To further elucidate the mechanism underlying the unexpected formation of **12** from **8d**, DFT calculations were performed (Figure 6). Protonation of **8d** affords **8d‐H^+^**, in which the proton resides on the terminal nitrogen atom of the azide. The geometry of this intermediate reveals an alignment of the azide π‐system with the C─B bond, predisposing the system for 1,2‐migration of the BMIDA moiety onto the azide through **TS1‐B**, with the Gibbs free energy of activation of 21.2 kcal/mol. Key structural features of **TS1‐B** include a C─N bond‐forming distance of 2.59 Å and a C─B bond‐breaking distance of 2.03 Å, leading to the formation of intermediate **8d‐INT1** in an overall exergonic process (Δ*G* = −37.8 kcal/mol). NBO analysis of this transition state confirmed strong donation from the C═N π‐system into the boron p‐orbital (π_C═N_ → B, *E*_INT_ = 267.5 kcal/mol), indicative of BMIDA acting as a proton analogue. Concomitantly, HOMO density is localized in‐phase across the C─N─B linkage, supporting a concerted 1,2‐boryl shift. This intermediate then undergoes nucleophilic trapping by the enol silane to form triazine **14**, or alternatively, hydrolysis followed by oxidation with triplet oxygen and hydrogen atom transfer (Δ*G* = −15.0 kcal/mol, see Supporting Information for details) to produce formaldehyde and azidoboronate **12**.

**FIGURE 7:**
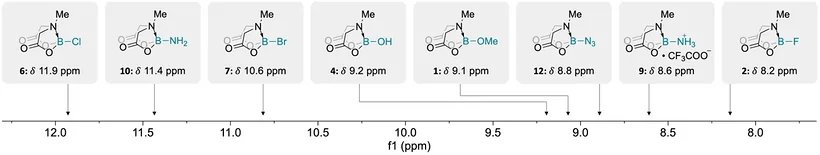
^11^B NMR table of compounds with boron‐heteroatom linkages (CD_3_CN, room temperature).

As a collection of boron‐heteroatom linkages‐containing compounds was established, we have compiled the ^11^B NMR chemical shifts table describing the species reported in this article (Figure 7). Boryl halides **6** and **7** have the most deshielded nuclei in the region of 10–12 ppm, whereas fluoroboronate **2** has a chemical shift of 8.2 ppm. Hydroxyboronate **4** and methoxyboronate **1** show similar peaks around 9 ppm. While the chemical shifts of **12** and **9** are in the same region (8–9 ppm), the peak of free aminoboronate **10** is downfield at 11.4 ppm. All ^11^B NMR chemical shifts are consistent with tetracoordinate boron‐containing compounds.

## 3 Conclusion

In summary, as part of a long‐standing effort to exert kinetic control over labile boron‐heteroatom bonds, we have come across rare examples of water‐stable boron‐heteroatom linkages. Despite the hemilability of BMIDA's B─N bond, surprising levels of kinetic stability can be achieved. Depending on the structure under consideration, acid‐promoted migration of BMIDA was also found to produce compounds with boron bound to nitrogen in different oxidation states. The formation of azidoboronate over aminoboronate with the expectedly low energy path to nitrogen elimination is a particularly interesting consequence of kinetic stabilization afforded by the MIDA group. The corresponding azides can now be used to make a wide range of molecules that feature BNP linkages under mild conditions. The formation of the boron‐containing triazine with surprising stability to aqueous workup and purification should open a new avenue in azide chemistry. It is difficult to imagine an alternative route to this reduced species using hydrogenation. We are not aware of reports that document the partial reduction of organic or inorganic azides. An additional practical significance of our work lies in the discovery of the first monomeric building block that features B─NH_2_ linkage. The amine nucleophilicity in compounds of this kind should now be examined in many areas.

## Author Contributions

**Alina Trofimova**: methodology, investigation, data curation, conceptualization, validation, formal analysis, writing – review and editing, writing – original draft, visualization. **Ben Zhen Huang**: methodology, validation, investigation, writing – review and editing, data curation, visualization, formal analysis. **Harjeet S. Soor**: conceptualization, methodology, data curation. **Brandon White**: software, visualization. **Aleksandra Holownia**: methodology. **Yury Lebedev**: methodology. **Alan J. Lough**: data curation. **Travis Dudding**: funding acquisition, project administration, resources, and supervision. **Andrei K. Yudin**: funding acquisition, writing – original draft, writing – review and editing, project administration, resources, supervision, conceptualization.

## Conflicts of Interest

The authors declare no conflicts of interest.

## Supporting information




**Supporting File**: The authors have cited additional references within the Supporting Information [60, 61, 62, 63, 64, 65, 66, 67, 68, 69, 70].

## Data Availability

The data that support the findings of this study are available on request from the corresponding author. The data are not publicly available due to privacy or ethical restrictions.
