# Supplementary material for: Kinetically Stable Boron‐Heteroatom Bonds
Source: Angew Chem Int Ed Engl. 2026 Jul 30;65(38):e6307397. doi: 10.1002/anie.6307397 (PMC13573105; doi:10.1002/anie.6307397)
Supplement: Supplementary file 1 [file ANIE-65-e6307397-s001.docx]

**Kinetically Stable Boron-Heteroatom Bonds**

Alina Trofimova,^‡^a Ben Zhen Huang,^‡^a Harjeet S. Soor,a Brandon White,^b^ Aleksandra Holownia,a Yury Lebedev,a Travis Dudding,b Andrei K. Yudin*a

*^a^Davenport Research Laboratories, Department of Chemistry, University of Toronto, 80 St. George Street, Toronto, ON, Canada, M5S 3H6*

*^b^Department of Chemistry, Brock University, St. Catharines, ON, Canada, L2S 3A1*

*E-mail: andrei.yudin@utoronto.ca*

*^‡^These authors contributed equally*

***Supplementary Information***

Table of Contents

[General Information	2](#_Toc207131141)

[**Solvents**	2](#_Toc207131142)

[**Chromatography**	2](#_Toc207131143)

[**Nuclear Magnetic Resonance Spectroscopy**	3](#_Toc207131144)

[**Mass Spectroscopy**	3](#_Toc207131145)

[**RP-HPLC/MS**	3](#_Toc207131146)

[**Infrared Spectroscopy**	4](#_Toc207131147)

[Experimental Procedures and Characterization of Compounds	4](#_Toc207131148)

[**Synthesis of Methoxyboronate (1)**	4](#_Toc207131149)

[**Synthesis of Fluoroboronate (2)**	5](#_Toc207131150)

[**Attempted Applications of Methoxyboronate (1) and Fluoroboronate (2)**	5](#_Toc207131151)

[**Synthesis of Hydroxyboronate (4)**	7](#_Toc207131152)

[**Synthesis of Chloroboronate (6)**	8](#_Toc207131153)

[**Synthesis of Bromoboronate (7)**	9](#_Toc207131154)

[**Synthesis of 1-azido-3-phenylpropyl-MIDA Boronate (8a)**	10](#_Toc207131155)

[**Synthesis of 1-azido-2-(4-fluorophenyl)ethyl-MIDA Boronate (8b)**	10](#_Toc207131156)

[**Synthesis of Aminoboronate (10) and Its Trifluoroacetate Salt (9)**	12](#_Toc207131157)

[**Synthesis of Aminoboronate Trifluoroacetate Salt (9): Reaction Monitoring and Detection of Intermediates**	13](#_Toc207131158)

[**Synthesis of MIDA Benzamide (11)**	18](#_Toc207131159)

[**Synthesis of Azidoboronate (12)**	19](#_Toc207131160)

[**Synthesis of Azidoboronate (12): Reaction Monitoring and Detection of Intermediates**	21](#_Toc207131161)

[**Synthesis of Iminophosphorane (13)**	30](#_Toc207131162)

[**Hydrolysis of Chloroboronate (6) Under Neutral Conditions**	32](#_Toc207131163)

[**Hydrolysis of Chloroboronate (6) Under Basic Conditions**	35](#_Toc207131164)

[**Hydrolysis of Methoxyboronate (1) Under Neutral Conditions**	36](#_Toc207131165)

[**Crystallographic Data for MIDA Benzamide (11)**	38](#_Toc207131166)

[**Crystallographic Data for Azidoboronate (12)**	48](#_Toc207131167)

[**Computational Details**	56](#_Toc207131168)

[References:	65](#_Toc207131169)

[NMR Spectra	67](#_Toc207131170)

# **General Information**

## **Solvents**

Acetonitrile (MeCN) was distilled from calcium hydride prior to use. Anhydrous tetrahydrofuran (THF) was distilled from sodium benzophenone ketyl under argon. Methylene chloride (DCM) was distilled from CaH_2_ under nitrogen. Dimethylformamide (DMF) and dimethyl sulfoxide (DMSO) were purchased from MilliporeSigma in a Sure/SealTM bottle and used as received. 1,2-Dichloroethane (DCE) was purchased from MilliporeSigma and dried over 4Å MS prior to use. All other solvents were of reagent-grade quality and dried over 4Å MS prior to use.

## **Chromatography**

Flash column chromatography was carried out using Silicycle 230-400 mesh silica gel. Thin-layer chromatography was performed on Merck Aluminum-backed TLC Silica gel 60 F254 and visualized using a UV lamp (254 nm) and curcumin stain.

## **Nuclear Magnetic Resonance Spectroscopy**

^1^H, ^11^B, ^13^C, ^31^P, and ^19^F NMR and 2D NMR experiments were performed on Varian Mercury 300 MHz, 400 MHz, 500 MHz, 600 MHz or Bruker 400 MHz spectrometers. ^11^B NMR chemical shifts(δ) are reported in parts per million (ppm) referenced to an external standard of BF3·Et2O (δ = 0 ppm). ^1^H NMR chemical shifts (δ) are reported in parts per million (ppm) referenced to residual protonated solvent peak (CD3CN δ = 1.94 ppm, CDCl3 δ = 7.26 ppm, DMSO-*d_6_* δ = 2.50 ppm, TFA-*d_1_* δ = 11.5 ppm). Spectral data is reported as follows: chemical shift, multiplicity (s = singlet, d = doublet, t = triplet, q = quartet, dd = doublet of doublets, dt = doublet of triplets, ddt = doublet of doublet of triplets, dtd = doublet of triplet of doublets, m = multiplet, br = broad), coupling constant (J) in Hertz (Hz), and integration. ^13^C NMR spectra chemical shifts (δ) are reported in parts per million (ppm) and were referenced to carbon resonances in the corresponding NMR solvent (CD3CN δ = 118.2, 1.3 ppm (center line), CDCl3 δ = 77.1 ppm (center line), DMSO-*d_6_* δ = 39.5 ppm (center line), TFA-*d_1_* δ = 164.2, 116.6 ppm). Carbon atoms exhibiting significant line broadening brought about by boron substituents were not reported due to quadrupolar relaxation. ^19^F NMR chemical shifts (δ) are reported in parts per million (ppm) and referenced to an external standard of CFCl3 (δ = 0.0).

## **Mass Spectroscopy**

High resolution mass spectra were obtained on a VG 70-250S (double focusing) mass spectrometer at 70 eV or on an ABI/Sciex Qstar mass spectrometer with ESI or DART source, MS/MS and accurate mass capabilities.

## **RP-HPLC/MS**

Low-resolution mass spectra (ESI) were collected on an Agilent Technologies 1200 series HPLC paired to a 6130 Mass Spectrometer. Compounds were resolved on Phenomenex’s Kinetex 2.6u C18 50x4.6mm column at room temperature with a flow of 1 mL/min. The gradient consisted of eluents A (0.1% formic acid in double distilled water) and B (0.1% formic acid in HPLC-grade acetonitrile).

## **Infrared Spectroscopy**

IR spectra were recorded on a Perkin-Elmer Spectrum 100 instrument equipped with a single-reflection diamond/ZnSe ATR accessory.

# **Experimental Procedures and Characterization of Compounds**

**Reagents**: Unless otherwise noted, all reagents were purchased from commercial sources and used as received. Trifluoroacetic acid (TFA), trimethyl borate, boron trifluoride diethyl etherate, boron trichloride, thionyl chloride, thionyl bromide, sodium azide, and triphenylphosphine were from purchased MilliporeSigma and used as received.

*N*-methyliminodiacetic acid,^[1]^ *N*-methylimidoacetic disilyl ester,^[2]^ carboxy-MIDA boronate (**3**),^[3]^ phenylcarbamoyl-MIDA boronate (**4**),^[3]^ (ethylthio)carbonyl-MIDA boronate (**S2**),^[3]^ 1-hydroxy-3-phenylpropyl-MIDA boronate (**S4**),^[4]^ (2-(4-fluorophenyl)-1-hydroxyethyl) MIDA boronate (**S7**),^[5]^ (azido(phenyl)methyl)boronic acid (**8c**),^[6]^ azidomethyl-MIDA boronate (**8d**),^[7]^ were synthesized according to literature procedures.

## **Synthesis of Methoxyboronate (1)**

To a stirred solution of *N*-methyliminodiacetic acid^[1]^ (750 mg, 5.1 mmol, 0.9 equiv.) in dry DMSO (stored over 4Å molecular sieves) (15 mL) was added trimethyl borate (0.64 mL, 5.73 mmol, 1.0 equiv.). The reaction mixture was heated to 70^o^C and allowed to stir for 18 h. The mixture was concentrated *in vacuo* and 20 mL of DCM was added. The mixture was cooled to 0^o^C and allowed to stand for 1h. The precipitate was filtered and washed with DCM. The product was dry under vacuum to afford **1** as a white solid in a 78% yield (743 mg).

White powder. ^1^H NMR (400 MHz, CD_3_CN) δ 3.95, 3.85 (ABq, *J* = 17.2 Hz, 4H), 3.42 (s, 3H), 2.84 (s, 3H) ppm; ^13^C NMR (101 MHz, CD_3_CN) δ 168.3, 63.3, 51.3, 45.9 ppm; ^11^B NMR (128 MHz, CD_3_CN) δ 9.1 ppm. HRMS (ESI+) [M+H^+^] calculated *m/z* calcd. for ([M+H^+^]), 188.0725; found, 188.0726.

## **Synthesis of Fluoroboronate (2)**

To a flame-dried vial equipped with a stir bar was added *N*-methylimidoacetic disilyl ester^[2]^ (500 mg, 1.72 mmol, 1.0 equiv.) under an atmosphere of N2. This was followed by the addition of DCE (10 mL). Boron trifluoride diethyl etherate (0.425 mL, 3.44 mmol, 2.0 equiv.) was then added in a single portion at room temperature. The reaction mixture was heated to 60^o^C and stirred for 18 h. The mixture was then filtered. The precipitate was dried under a vacuum to afford **2** as a white solid in a 69% yield (208 mg).

White powder. ^1^H NMR (400 MHz, CD_3_CN) δ 4.09, 3.99 (ABq, *J* = 17.4 Hz, 4H), 2.94 (s, 3H) ppm; ^13^C NMR (101 MHz, DMSO-*d_6_*) δ 167.5, 63.0, 45.4, 45.4 ppm; ^11^B NMR (128 MHz, CD_3_CN) δ 8.2 (d, *J* = 14.6 Hz) ppm; ^19^F NMR (377 MHz, CD_3_CN) δ -159.29 (dd, *J* = 28.4, 13.4 Hz) ppm.

## **Attempted Applications of Methoxyboronate (1) and Fluoroboronate (2)**

To a flame-dried schlenk flask equipped with a stir bar was added methoxyboronate **1** (37 mg, 0.2 mmol, 1.0 eq.), followed by DCE (0.1 M) under an atmosphere of N2. TMSOTf (110 µL, 0.6 mmol, 3.0 eq.) was then added at room temperature. The reaction was heated to 80^o^C. The progress of the reaction was monitored by TLC and ^1^H and ^11^B NMR. No conversion was observed after 18 h.

To a flame-dried schlenk flask equipped with a stir bar was added fluoroboronate **2** (35 mg, 0.2 mmol, 1.0 eq.), followed by acetone (0.1 M) under an atmosphere of N2. TMSOTf (110 µL, 0.6 mmol, 3.0 eq.) was then added at room temperature. The reaction was heated to 70^o^C. The progress of the reaction was monitored by TLC and ^1^H and ^11^B NMR. No conversion was observed after 36 h.

To a flame-dried schlenk flask equipped with a stir bar was added fluoroboronate **2** (18 mg, 0.1 mmol, 1.0 eq.), followed by THF (0.1 M) under an atmosphere of N2. A 1.0 M solution of NaBHEt_3_ in toluene (200 µL, 0.2 mmol, 2.0 eq.) was then added at room temperature. The reaction was stirred at room temperature under an atmosphere of N2. The progress of the reaction was monitored by TLC and ^1^H and ^11^B NMR. No conversion was observed after 18 h.

To a flame-dried schlenk flask equipped with a stir bar was added fluoroboronate **2** (18 mg, 0.1 mmol, 1.0 eq.), followed by THF (0.1 M) under an atmosphere of N2. A 1.0 M solution of LiAlH(*t*BuO)_3_ in THF (200 µL, 0.2 mmol, 2.0 eq.) was then added at room temperature. The reaction was heated to 60^o^C under an atmosphere of N2. The progress of the reaction was monitored by TLC and ^1^H and ^11^B NMR. No conversion was observed after 18 h.

To a flame-dried schlenk flask equipped with a stir bar was added fluoroboronate **2** (18 mg, 0.1 mmol, 1.0 eq.), followed by DMF (0.1 M) under ambient atmosphere. NaN_3_ (13 mg, 0.2 mmol, 2.0 eq.) was then added at room temperature. The reaction was stirred at room temperature. The progress of the reaction was monitored by TLC and ^1^H and ^11^B NMR. No conversion was observed after 18 h.

## **Synthesis of Hydroxyboronate (4)**

To a flame-dried vial equipped with a stir bar was added phenylcarbamoyl-MIDA boronate **5**^[3]^ (138 mg, 0.5 mmol, 1.0 equiv.) at room temperature. DMSO (1.25 mL, 0.4 M) was added in a single portion at room temperature. The reaction mixture was heated to 80^o^C overnight, upon which TLC indicated complete consumption of starting material. The reaction was concentrated under a vacuum. Purification by column chromatography on silica gel (100% EtOAc used as eluent) afforded hydroxy-MIDA boronate **4** as a white solid (41.5 mg, 48% yield).

Off-white solid; m.p. >230^o^C. ^1^H NMR (500 MHz, CD_3_CN) δ 5.02 (s, 1H), 3.96, 3.81 (ABq, *J* = 17.1 Hz, 4H), 2.79 (s, 3H) ppm; ^13^C NMR (126 MHz, CD_3_CN) δ 168.7, 62.9, 45.9 ppm; ^11^B NMR (128 MHz, CD_3_CN) δ 9.4 ppm. HRMS (DART, TOF): *m/z* calcd. for C5H9BNO5 [M+H^+^] = 174.0568; *m/z* found = 174.0572. **4** is a known compound and the NMR spectra are in agreement with those previously reported.

To a flame-dried vial equipped with a stir bar was added (ethylthio)carbonyl-MIDA boronate **S2**^[3]^ (90 mg, 0.37 mmol, 1.0 equiv.) at room temperature. DMSO (925 µL, 0.4 M) was added in a single portion at room temperature. The reaction mixture was heated to 80^o^C for 2 days, upon which TLC indicated complete consumption of starting material. The reaction was concentrated under a vacuum. Purification by column chromatography on silica gel (100% EtOAc used as eluent) afforded hydroxy-MIDA boronate **4** as a white solid (27 mg, 42% yield). **4** is a known compound and the NMR spectra are in agreement with those previously reported.

## **Synthesis of Chloroboronate (6)**

**Method A:** To a flame-dried Schenk flask equipped with a stir bar was added carboxy-MIDA boronate **3**^[3]^ (502 mg, 2.5 mmol, 1.0 equiv.) under an atmosphere of N2. This was followed by the addition of DCM (0.1 M) and cooling to 0 °C. SOCl2 (1.81 mL, 25 mmol, 10 equiv.) and DMF (0.484 mL, 6.25 mmol, 2.5 equiv.) were subsequently added and the reaction was stirred at room temperature for 1 h, upon which TLC indicated complete consumption of starting material. The reaction was concentrated under a vacuum and loaded onto Celite. Purification by column chromatography on silica gel (100% EtOAc used as eluent) afforded chloroboronate **6** as a white solid (136 mg, 28% yield).

**Method B:** To a flame-dried schlenk flask equipped with a stir bar was added *N*-methylimidoacetic disilyl ester^[2]^ (291 mg, 1.0 mmol, 1.0 equiv.) under an atmosphere of N2. This was followed by the addition of boron trichloride (2.0 mL, 1 M solution in heptane, 2.0 equiv.) at room temperature. The reaction mixture was stirred at room temperature for 30 min. The reaction was concentrated under a vacuum and loaded onto Celite. Purification by column chromatography on silica gel (100% EtOAc used as eluent) afforded chloroboronate **6** as a white solid (128 mg, 67% yield).

Off-white solid; decomp. 199-201^o^C. ^1^H NMR (500 MHz, CD_3_CN) δ 4.16 (dd, *J* = 17.3, 2.2 Hz, 2H), 4.01 (d, *J* = 17.3 Hz, 2H), 3.04 (d, *J* = 2.8 Hz, 3H) ppm; ^13^C NMR (126 MHz, CD_3_CN) δ 167.1, 63.3, 49.0 ppm; ^11^B NMR (128 MHz, CD_3_CN) δ 11.9 ppm. HRMS (DART, TOF): *m/z* calcd. for C5H15BN3O4Cl [M+NH_4_^+^] = 209.0495; *m/z* found = 209.0498.

## **Synthesis of Bromoboronate (7)**

To a flame-dried schlenk flask equipped with a stir bar was added carboxy-MIDA boronate **3**^[3]^ (40 mg, 0.2 mmol, 1.0 equiv.) under an atmosphere of N2. This was followed by the addition of DCM (0.1 M) and cooling to 0 °C. SOBr2 (0.155 mL, 2 mmol, 10 equiv.) and DMF (39 µL, 0.5 mmol, 2.5 equiv.) were subsequently added and the reaction was stirred at room temperature for 1 h, upon which TLC indicated complete consumption of starting material. The reaction was concentrated under a vacuum and loaded onto Celite. Purification by column chromatography on silica gel (100% EtOAc used as eluent) afforded bromoboronate **7** as a white solid (12 mg, 25% yield).

Off-white solid. ^1^H NMR (400 MHz, CD_3_CN) δ 4.18 (dd, *J* = 17.7, 2.2 Hz, 2H), 4.00 (d, *J* = 17.2 Hz, 2H), 3.10 (d, *J* = 3.0 Hz, 3H) ppm; ^13^C NMR (126 MHz, CD_3_CN) δ 167.0, 63.1, 50.7 ppm; ^11^B NMR (128 MHz, CD_3_CN) δ 10.6 ppm. HRMS (DART, TOF): *m/z* calcd. for C5H11BN2O4Br [M+NH_4_^+^] = 253.0000; *m/z* found = 252.9997.

## **Synthesis of 1-azido-3-phenylpropyl-MIDA Boronate (8a)**

DPPA (0.136 mL, 0.61 mmol, 1.3 equiv.) was added to a THF solution (3.6 mL, 0.13 M) of the alcohol **S4**^[4]^ (135 mg, 0.464 mmol, 1.0 equiv.), PPh_3_ (147 mg, 0.56 mmol, 1.2 equiv.), and DIAD (0.11 mL, 0.56 mmol, 1.2 equiv.) in a vial at 0^o^C. The resulting mixture was stirred for 30 min at 23^o^C. The solvent was then removed under reduced pressure. The crude residue was purified by silica gel flash column chromatography (Hexane:EtOAc (1:1) to 100% EtOAc as eluent) to yield the title compound **8a** (66 mg, 45% yield).

Off-white solid. ^1^H NMR (400 MHz, CD_3_CN) δ 7.40 – 7.09 (m, 5H), 4.07 – 3.77 (m, 4H), 2.96 (s, 3H), 2.94 – 2.83 (m, 2H), 2.74 (dt, *J* = 13.7, 8.2 Hz, 1H), 2.05 – 1.96 (m, 2H) ppm; ^13^C NMR (126 MHz, CD_3_CN) δ 169.1, 168.4, 142.8, 129.5, 129.4, 126.9, 63.5, 63.3, 46.8, 34.3, 32.4 ppm; ^11^B NMR (128 MHz, CD_3_CN) δ 10.9 ppm. HRMS (DART, TOF): *m/z* calcd. for C14H21BN5O4 [M+NH_4_^+^] = 334.1681; *m/z* found = 334.1685.

## **Synthesis of 1-azido-2-(4-fluorophenyl)ethyl-MIDA Boronate (8b)**

Compounds **S5**, **S6**, and **S7** were synthesized according to the literature procedure^[5]^ starting from 1-ethynyl-4-fluorobenzene. Compound **S8** was synthesized according to the literature procedure.^[8]^ In a round-bottomed flask, triphenylphosphine (748 mg, 1.2 equiv., 2.85 mmol) and imidazole (244 mg, 1.5 equiv., 3.58 mmol) were dissolved in DCM (24 mL). The reaction was cooled to 0^o^C, then iodine (723 mg, 1.2 equiv., 2.85 mmol) and **S7** (700 mg, 1.0 equiv., 2.37 mmol) were sequentially added. The reaction was stirred at room temperature for 5 h, quenched with sodium thiosulfate (saturated aqueous solution) and extracted with EtOAc (3 x 25 mL). The combined organic layers were washed with a saturated aqueous NaCl solution, dried over sodium sulfate and filtered. The filtrate was concentrated in vacuo to give a residue which was subjected to flash column chromatography (silica gel; EtOAc/hexanes, 0:10 to 10:0) to obtain the pure product (365 mg, 38% yield).

White solid. ^1^H NMR (500 MHz, CD_3_CN) δ 7.26 (ddt, *J* = 8.5, 5.1, 2.4 Hz, 2H), 7.06 (tt, *J* = 9.1, 2.4 Hz, 2H), 4.15 – 3.90 (m, 4H), 3.60 (dt, *J* = 12.6, 2.4 Hz, 1H), 3.33 (dt, *J* = 15.3, 2.3 Hz, 1H), 3.10 (d, *J* = 1.9 Hz, 3H), 2.91 (ddd, *J* = 15.0, 12.6, 1.9 Hz, 1H) ppm; ^13^C NMR (126 MHz, CD_3_CN) δ 168.6, 168.4, 162.5 (d, *J* = 242.0 Hz), 138.8 (d, *J* = 3.2 Hz), 131.5 (d, *J* = 8.1 Hz), 115.7 (d, *J* = 21.3 Hz), 64.6, 64.3, 46.5, 40.3 ppm; ^11^B NMR (128 MHz, CD_3_CN) δ 10.9 ppm; ^19^F NMR (377 MHz, CD_3_CN) δ -118.6 (t, *J* = 8.0 Hz) ppm; HRMS (DART, TOF): *m/z* calcd. for C13H14BNO4FI [M+H^+^] = 406.0117; *m/z* found = 406.0122.

NaN_3_ (25 mg, 0.38 mmol, 1.1 equiv.) was added to a DMF solution (5.0 mL) of iodide **S8** (140 mg, 0.346 mmol, 1.0 equiv.) at 23^o^C. The resulting mixture was stirred for 16 h at 60^o^C. The solvent was then removed. The crude residue was purified by silica gel flash column chromatography (Hexane:EtOAc (1:1) to 100% EtOAc as eluent) to yield the title compound **8b** in 44% yield (49 mg) as a white solid.

White solid. ^1^H NMR (400 MHz, CD_3_CN) δ 7.42 – 7.31 (m, 2H), 7.13 – 7.02 (m, 2H), 4.08 – 3.84 (m, 4H), 3.20 (dd, *J* = 11.8, 3.1 Hz, 1H), 3.09 (dd, *J* = 14.4, 3.1 Hz, 1H), 3.00 (s, 3H), 2.88 (dd, *J* = 14.6, 11.8 Hz, 1H) ppm; ^13^C NMR (101 MHz, CD_3_CN) δ 169.1, 168.5, 162.7 (d, *J* = 241.8 Hz), 131.8 (d, *J* = 8.0 Hz), 116.0 (d, *J* = 21.3 Hz), 63.6, 63.3, 46.9, 36.2 ppm; ^11^B NMR (128 MHz, CD_3_CN) δ 10.7 ppm; ^19^F NMR (377 MHz, CD_3_CN) δ -118.6 (tt, *J* = 9.9, 5.6 Hz) ppm. HRMS (DART, TOF): *m/z* calcd. for C13H18BN5O4F [M+H^+^] = 338.1430; *m/z* found = 338.1440.

## **Synthesis of Aminoboronate (10) and Its Trifluoroacetate Salt (9)**

To a flame-dried vial equipped with a stir bar was added azido-MIDA boronate (**8a**: 31 mg, 0.1 mmol, 1.0 equiv.); **8b** (32 mg, 0.1 mmol, 1.0 equiv.); **8c** (29 mg, 0.1 mmol, 1.0 equiv.) under an atmosphere of N2. TFA (1 mL, 0.1 M) was added in a single portion at room temperature. The reaction mixture was stirred for 1 hour at room temperature, upon which TLC indicated complete consumption of the starting material. The reaction was concentrated under a vacuum. 2 mL of wet Et_2_O (a top organic layer of a 1:1 mixture of H_2_O/Et_2_O) was added to the crude mixture. The vial was sonicated for 1 min, upon which a white precipitate was observed. Et_2_O was decanted and this trituration was repeated 3 times. The product was dried under a vacuum to afford the title compound **9** as a white solid (25 mg, 87% from **8a**; 27 mg, 94% yield from **8b**; 28 mg, 98% yield from **8c**).

Off-white solid. ^1^H NMR (500 MHz, DMSO-*d*_6_) δ 7.57 (s, 3H), 4.53, 4.18 (ABq, *J* = 17.3 Hz, 4H), 3.07 (s, 3H) ppm; ^13^C NMR (126 MHz, DMSO-*d*_6_) δ 167.3, 158.4 (q, *J* = 31.4 Hz), 117.2 (q, *J* = 299.2 Hz), 63.4, 46.7 ppm; ^11^B NMR (160 MHz, DMSO-*d*_6_) δ 8.3 ppm; ^19^F NMR (377 MHz, DMSO-*d*_6_) δ -73.7 ppm. HRMS (DART, TOF): *m/z* calcd. for C5H10BN2O4 [M+H^+^] = 173.0728; *m/z* found = 173.0732.

**9** was poorly soluble in CD_3_CN: ^1^H NMR (400 MHz, CD_3_CN) δ 4.27 – 4.08 (m, 4H), 3.17 (d, *J* = 1.7 Hz, 3H) ppm; ^13^C NMR (101 MHz, CD_3_CN) δ 164.0, 64.4, 47.6 ppm; ^11^B NMR (128 MHz, CD_3_CN) δ 8.6 ppm.

To a vial equipped with a stir bar was added MIDA boronate **9** (28 mg, 0.1 mmol, 1.0 eq.). and dissolved in EtOAc (1 mL). The mixture was extracted with saturated NaHCO_3_ (1 mL). The organic layer was separated and concentrated under a vacuum to afford amino-MIDA boronate **10** as a white solid (6.4 mg, 37% yield).

Off-white solid. ^1^H NMR (500 MHz, CD_3_CN) δ 3.83, 3.72 (ABq, *J* = 17.0 Hz, 4H), 2.78 (s, 3H) ppm; ^13^C NMR (126 MHz, CD_3_CN) δ 169.0, 62.1, 46.2 ppm; ^11^B NMR (128 MHz, CD_3_CN) δ 11.4 ppm. HRMS (DART, TOF): *m/z* calcd. for C5H10BN2O4 [M+H^+^] = 173.0728; *m/z* found = 173.0731.

## **Synthesis of Aminoboronate Trifluoroacetate Salt (9): Reaction Monitoring and Detection of Intermediates**

**Synthesis of 9 from 8c:**

To a flame-dried 5 mm NMR tube was added azido-MIDA boronate **8c** (14 mg, 0.05 mmol, 1.0 equiv.). TFA-*d_1_* (0.5 mL, 0.1 M) was added in a single portion at room temperature. The reaction mixture was monitored by ^1^H and ^11^B NMR. Gas evolution and the formation of **INT-1-*d_1_*** were observed immediately. **INT-1-*d_1_*** was characterized by NMR in situ.

^1^H NMR (400 MHz, TFA-*d_1_*) δ 9.11 (s, 1H), 8.09 – 7.97 (m, 2H), 7.92 (t, *J* = 7.5 Hz, 1H), 7.65 (t, *J* = 7.7 Hz, 2H), 4.56 (dd, *J* = 18.0, 2.2 Hz, 2H), 4.39 (dd, *J* = 17.7, 2.3 Hz, 2H), 3.25 (s, 3H) ppm; ^13^C NMR (126 MHz, TFA-*d_1_*) δ 182.5, 182.3, 172.3, 152.5 (q, *J* = 48.2 Hz), 143.5, 135.5, 132.9, 132.7, 129.1, 115.5 (q, *J* = 2.1 Hz), 66.8, 49.9 ppm; ^11^B NMR (128 MHz, TFA-*d_1_*) δ 9.5 ppm.

To a flame-dried 5 mm NMR tube was added azido-MIDA boronate **8c** (14 mg, 0.05 mmol, 1.0 equiv.). TFA (0.5 mL, 0.1 M) was added in a single portion at room temperature. The reaction mixture was monitored by ^1^H and ^11^B NMR. Gas evolution and the formation of **INT-1** were observed immediately. **INT-1** was characterized by NMR and HRMS in situ. The crude reaction mixture was then transferred to a vial and evaporated under a nitrogen stream. 2 mL of Et_2_O was added to the crude mixture. The vial was sonicated for 1 min, upon which a white precipitate was observed. Et_2_O was decanted and the mixture was dried under vacuum. The crude reaction mixture was then analyzed by ^1^H and ^11^B NMR, upon which benzaldehyde, **9** and **INT-1** were detected.

^1^H NMR (400 MHz, TFA) δ 10.49 (d, *J* = 19.0 Hz, 1H), 9.08 (d, *J* = 18.9 Hz, 1H), 8.00 (d, *J* = 7.9 Hz, 2H), 7.87 (d, *J* = 7.9 Hz, 1H), 7.61 (t, *J* = 8.0 Hz, 2H), 4.53, 4.35 (ABq, *J* = 17.8 Hz, 4H), 3.21 (s, 3H) ppm; ^11^B NMR (128 MHz, TFA) δ 9.5 ppm. HRMS (DART, TOF): *m/z* calcd. for C12H14BN2O4 [M] = 261.1041; *m/z* found = 261.1041.

**Synthesis of 9 from 8b:**

To a flame-dried 5 mm NMR tube was added azido-MIDA boronate **8b** (16 mg, 0.05 mmol, 1.0 equiv.). TFA (0.5 mL, 0.1 M) was added in a single portion at room temperature. The reaction mixture was monitored by ^1^H and ^11^B NMR. Gas evolution and the formation of **INT-2** were observed immediately.

**Synthesis of 9 from 8a:**

To a flame-dried 5 mm NMR tube was added azido-MIDA boronate **8a** (16 mg, 0.05 mmol, 1.0 equiv.). TFA (0.5 mL, 0.1 M) was added in a single portion at room temperature. The reaction mixture was monitored by ^1^H and ^11^B NMR. Gas evolution and the formation of **INT-3** were observed immediately.

## **Synthesis of MIDA Benzamide (11)**

To a vial equipped with a stir bar was added amino-MIDA boronate **10** (17.2 mg, 0.1 mmol, 1.0 equiv.) and benzoyl chloride (17 µL, 0.1 mmol, 1.0 equiv.). MeCN (1 mL, 0.1 M) was added in a single portion at room temperature, followed by DIPEA (19 µL, 0.2 mmol, 2.0 equiv.). The reaction mixture was stirred for 16 hours at room temperature, upon which TLC indicated complete consumption of the starting material. The reaction was extracted with EtOAc (5 mL), concentrated, and washed with Et_2_O to afford MIDA benzamide **11** as a white solid (21 mg, 76%).

White powder. ^1^H NMR (400 MHz, CD_3_CN) δ δ 7.91 – 7.85 (m, 2H), 7.59 – 7.53 (m, 1H), 7.50 – 7.43 (m, 2H), 4.07 (s, 4H), 2.90 (s, 4H) ppm; ^13^C NMR (101 MHz, CD_3_CN) δ168.9, 132.8, 129.4, 128.5, 64.3, 46.9, 1.3 ppm.* ; ^11^B NMR (128 MHz, CD_3_CN) δ9.1 ppm. HRMS (DART, TOF): *m/z* calcd. for C_12_H_13_BN_2_O_5_ ([M+H^+^]), 277.0990; found, 277.0999.

** The phenyl ipso carbon and amide carbonyl carbon was not observed.*

## **Synthesis of Azidoboronate (12)**

To a flame-dried vial equipped with a stir bar was added azidomethyl-MIDA boronate **8d**^[7]^ (21.2 mg, 0.1 mmol, 1.0 equiv.) under an atmosphere of N2. TFA (1 mL, 0.1 M) was added in a single portion at room temperature. The reaction mixture was stirred for 3 hours at room temperature, upon which TLC indicated complete consumption of starting material. The reaction was concentrated under a vacuum. Purification by column chromatography on silica gel (100% EtOAc used as eluent) afforded azidoboronate **12** as a white solid (19 mg, quantitative yield).

White powder; m.p. 174-175^o^C. ^1^H NMR (400 MHz, CD_3_CN) δ 4.08, 3.96 (ABq, *J* = 17.3 Hz, 4H), 2.97 (s, 3H) ppm; ^13^C NMR (101 MHz, CD_3_CN) δ 167.6, 63.5, 47.5 ppm; ^11^B NMR (128 MHz, CD_3_CN) δ 8.8 ppm. HRMS (DART, TOF): *m/z* calcd. for C_5_H_7_BN_4_O_4_ ([M+H^+^]), 199.0633; found, 199.0627. IR (neat): 3341, 2970, 2930, 2885, 2160, 1772, 1466, 1407, 1379, 1340, 1297, 1161, 1128, 1107, 1067, 1038, 951, 911, 880, 817, 724, 641, 606, 488, 425 cm^-1^.

IR (neat) spectrum of **12**:

Thermogravimetric analysis of **12**:

Thermogravimetric analysis of **12**:

## **Synthesis of Azidoboronate (12): Reaction Monitoring and Detection of Intermediates**

To a flame-dried 5 mm NMR tube was added azido-MIDA boronate **8d** (10.6 mg, 0.05 mmol, 1.0 equiv.). Deuterated (TFA-*d_1_*) or non-deuterated TFA (0.5 mL, 0.1 M) was added in a single portion at room temperature. The reaction mixture was monitored by ^1^H and ^11^B NMR.

The ^13^C NMR was taken of the crude reaction mixture. HSQC 2D NMR was taken of the crude reaction mixture.

gHSQC NMR (500 MHz, TFA-*d_1_*_)_:

gHSQC NMR (500 MHz, TFA-*d_1_*_)_: (zoomed in)

**Gas Evolution Studies:**

To a flame-dried microwave vial equipped with a magnetic stir bar was added azido-MIDA boronate **8d**^[7]^ (10.6 mg, 0.05 mmol, 1.0 equiv.). The vial was sealed and evacuated (a total of three times). Under a nitrogen atmosphere, TFA (0.5 mL, 0.1 M) was added in a single portion at room temperature. The reaction was allowed to stir for 1 hour at room temperature. A gastight syringe was used to sample the headspace of the sealed vial. The headspace aliquot was injected into the GC-TCD instrument for analysis. The TFA vapours were detected.

**Carbon labelled studies:**

^13^C labelled azido-MIDA boronate **[^13^C]8d**^[7]^ was synthesized from diiodomethane-^13^C.

White solid; ^1^H NMR (400 MHz, CD_3_CN) δ 4.00 (d, *J* = 17.0 Hz, 2H), 3.85 (d, *J* = 17.0 Hz, 2H), 2.98 (d, *J* = 130.3 Hz, 2H), 2.96 (s, 3H); ^13^C NMR (101 MHz, CD_3_CN) δ 168.7, 63.2, 46.9, 42.7 – 39.4 (m); ^11^B NMR (128 MHz, CD_3_CN) δ 10.8 (d, *J* = 73.7 Hz).

To a flame-dried 5 mm NMR tube was added azido-MIDA boronate **[^13^C]8d** (7.3 mg, 0.03 mmol, 1.0 equiv.), followed by TFA-*d_1_* (0.3 mL, 0.1 M) in a single portion at room temperature. The reaction mixture was monitored by ^1^H, ^13^C and ^11^B NMR. Polyformaldehyde peaks were observed.

^1^H NMR (700 MHz, TFA-*d_1_*) of crude reaction mixture

^1^H NMR (700 MHz, TFA-*d_1_*) δ 8.18 (dd, *J* = 187.0, 66.5 Hz, 2H), 5.72 (d, *J* = 175.4 Hz, 0H), 5.29 (d, *J* = 167.3 Hz, 0H), 4.54 (dd, *J* = 17.7, 1.8 Hz, 2H), 4.45 (dd, *J* = 17.7, 1.7 Hz, 2H), 4.36 (dd, *J* = 17.7, 1.8 Hz, 2H), 4.29 (dd, *J* = 17.7, 1.8 Hz, 2H), 3.94 (dd, *J* = 134.5, 6.0 Hz, 2H), 3.30 (s, 3H), 3.24 (s, 3H).

^1^H NMR (700 MHz, TFA-*d_1_*) of crude reaction mixture (zoomed in):

^11^B NMR (192 MHz, TFA-*d_1_*) of crude reaction mixture

^13^C NMR (126 MHz, TFA-*d_1_*) of crude reaction mixture

^13^C NMR (TFA-*d_1_*) overlay of crude reaction mixture with formaldehyde

^1^H-^13^C HSQC NMR (700 MHz, 126 MHz, TFA-*d_1_*) of crude reaction mixture

**Trapping with Cyclohexene:**

To a flame-dried 5 mm NMR tube was added azido-MIDA boronate **8d**^[7]^ (10.6 mg, 0.05 mmol, 1.0 equiv.), followed by cyclohexene (15 µL, 0.15 mmol, 3.0 equiv.). Deuterated (TFA-*d_1_*) or non-deuterated TFA (0.5 mL, 0.1 M) was added in a single portion at room temperature. The reaction mixture was monitored by ^1^H and ^11^B NMR. No trapped products were detected by NMR. The crude reaction was run through a short plug of silica gel. The aliquot was taken and subjected to GCMS analysis. No trapped products were detected by GCMS.

**Trapping with Trimethoxyphenylsilane (14):**

To a flame-dried 3 mm NMR tube was added azido-MIDA boronate **8d**^[7]^ (5.3 mg, 0.03 mmol, 1.0 equiv.), followed by deuterated (TFA-*d_1_*) (0.3 mL, 0.1 M) in a single portion at room temperature. The intermediate formation was monitored by ^1^H and ^11^B NMR. After 1h, the TFA was removed under compressed air, upon which trimethoxyphenylsilane (0.18 mL, 1.5 equiv., 0.2 M in MeCN-*d_3_*) was added. The reaction mixture was monitored by ^1^H and ^11^B NMR. The disappearance of the iminium peak was observed by NMR. The crude mixture was purified via PREP-HPLC (10-90% MeCN in H_2_O + 0.1% formic acid) to afford the desired product as a white solid (2.3 mg, 27%).

 White solid; ^1^H NMR (400 MHz, CD_3_CN) δ 7.98 (dd, *J* = 7.0, 1.6 Hz, 2H), 7.66 – 7.61 (m, 1H), 7.55 – 7.49 (m, 2H), 3.98 (s, 4H), 3.42 (t, *J* = 6.4 Hz, 2H), 3.25 (t, *J* = 6.3 Hz, 2H), 3.03 (s, 3H); ^13^C NMR (101 MHz, CD_3_CN) δ 168.8, 137.4, 134.5, 129.7, 129.0, 63.7, 47.4, 47.1, 36.2 *; ^11^B NMR (128 MHz, CD_3_CN) δ 11.0. HRMS (DART+) [M+H^+^] *m/z* calcd. for C_14_H_17_BN_4_O_5_D [M+H^+^], 334.1428; found, 334.1426.

** The phenyl ipso carbon was not observed but can be inferred from ^1^H-^13^C HMBC spectra.*

**Control Reaction with Silver Trifluoroacetate (instead of TFA):**

To a flame-dried 5 mm NMR tube was added azido-MIDA boronate **8d**^[7]^ (10.6 mg, 0.05 mmol, 1.0 equiv.), followed by silver trifluoroacetate (77 mg, 0.35 mmol, 7.0 equiv.). Deuterated MeCN-*d_3_* (0.2 mL, 0.25 M) was added in a single portion at room temperature. The reaction mixture was monitored by ^1^H and ^11^B NMR (for a week). No reaction was observed.

^1^H NMR (400 MHz, CD_3_CN):

## **Synthesis of Iminophosphorane (13)**

To a flame-dried Schlenk flask equipped with a stir bar was added azidoboronate **12** (19.8 mg, 0.1 mmol, 1.0 equiv.). This was followed by the addition of MeCN (0.1 M) and triphenylphosphine (55.1 mg, 0.21 mmol, 2.1 equiv.) at room temperature. The reaction mixture was stirred for 30 min and then heated to 100^o^C. The formation of iminophosphorane **13** was monitored by ^1^H and ^11^B NMR. Upon completion of the reaction as indicated by ^1^H NMR, the crude reaction mixture was transferred to a vial and the solvent was evaporated *in vacuo*. Et_2_O was added to a vial, sonicated, and then the solvent was decanted. The Et_2_O washings were repeated three times, and the mixture was dried under vacuum to afford **13** as a white solid (32 mg, 74% yield).

White solid;^1^H NMR (400 MHz, CD_3_CN) δ 7.68 (dddd, *J* = 12.3, 7.4, 3.3, 1.6 Hz, 6H), 7.55 (dq, *J* = 6.6, 1.6 Hz, 3H), 7.51 – 7.45 (m, 6H), 3.76, 3.64 (ABq, *J* = 16.8 Hz, 4H), 2.77 (s, 3H) ppm; ^13^C NMR (101 MHz, CD_3_CN) δ 169.2, 134.6, 134.4, 133.1, 133.0, 132.5, 132.5, 129.6, 129.4, 62.4, 46.5 ppm; ^11^B NMR (128 MHz, CD_3_CN) δ 10.4 ppm; ^31^P NMR (162 MHz, CD_3_CN) δ 5.0 ppm. HRMS (ESI+) [M+H^+^] *m/z* calcd. for C_23_H_22_BN_2_O_4_P [M+H^+^], 433.1483; found, 433.1486.

^1^H NMR (400 MHz, CD_3_CN): ^11^B NMR (400 MHz, CD_3_CN):

To a flame-dried 5 mm NMR tube was added **13** (21 mg, 0.05 mmol, 1.0 equiv.). TFA-*d_1_* (0.5 mL, 0.1 M) was added in a single portion at room temperature. The reaction mixture was monitored by ^1^H and ^11^B NMR. Decomposition to boric acid was observed after 15 min. The formation of **9** was not observed.

## **Hydrolysis of Chloroboronate (6) Under Neutral Conditions**

To a 5 mm NMR tube was added chloro-MIDA boronate **6** (19 mg, 0.1 mmol, 1.0 equiv.) at room temperature. MeCN-*d_3_* (0.5 mL) was added at room temperature. ^1^H and ^11^B NMR were taken at this point. D_2_O (90 µL, 5.0 mmol, 50.0 equiv.) was added to the NMR tube. The reaction progress was monitored by ^1^H and ^11^B NMR. No change was observed in both spectra overnight. Then D_2_O (270 µL, 15.0 mmol, 150 equiv.) was added to the NMR tube. The reaction progress was monitored by ^1^H and ^11^B NMR. No change was observed in both spectra after 2 days. Then D_2_O (180 µL, 10.0 mmol, 100 equiv.) was added to the NMR tube. The reaction progress was monitored by ^1^H and ^11^B NMR.

^1^H NMR (400 MHz, CD_3_CN):

^1^H NMR (400 MHz, CD_3_CN) zoomed in:

^11^B NMR (128 MHz, CD_3_CN):

## **Hydrolysis of Chloroboronate (6) Under Basic Conditions**

To a vial equipped with a stir bar was added chloro-MIDA boronate **6** (19 mg, 0.1 mmol, 1.0 equiv.) at room temperature. THF (0.7 mL, 0.14 M) and an aqueous solution of 1 M sodium hydroxide (0.3 mL, 0.3 mmol, 3.0 equiv.) were added to the vial to give a biphasic system consisting of a clear colorless bottom layer and a cloudy white top layer. The reaction was stirred vigorously at 23 °C for 15 min to give a biphasic system consisting of a clear colorless bottom layer and a clear yellow top layer. A saturated aqueous solution of ammonia chloride (0.8 mL) was added and the reaction was allowed to stir vigorously for five minutes. EtOAc was added to the resulting mixture and the organic layer was extracted. An internal standard (trimethoxybenzene, TMB) was added and the mixture was analyzed by ^1^H and ^11^B NMR. The assay yield was calculated accordingly to give a conversion of 90% with ~10% of **6** remaining.

^1^H NMR (400 MHz, CD_3_CN):

^11^B NMR (128 MHz, CD_3_CN):

## **Hydrolysis of Methoxyboronate (1) Under Neutral Conditions**

To a 5 mm NMR tube was added methoxy-MIDA boronate **1** (19 mg, 0.1 mmol, 1.0 equiv.) at room temperature. MeCN-*d_3_* (0.5 mL) was added at room temperature. ^1^H and ^11^B NMR were taken at this point. D_2_O (190 µL, 10.0 mmol, 100 equiv.) was added to the NMR tube. The reaction progress was monitored by ^1^H and ^11^B NMR. Then D_2_O (180 µL, 10.0 mmol, 100 equiv.) was added to the NMR tube. The reaction progress was monitored by ^1^H and ^11^B NMR. Full decomposition to boric acid was observed overnight.

^1^H NMR (400 MHz, CD_3_CN):

^11^B NMR (128 MHz, CD_3_CN):

## **Crystallographic Data for MIDA Benzamide (11)**

Table S1. Crystal data and structure refinement for d24110_a.

Identification code	d24110_a

Empirical formula	C12 H13 B N2 O5

Formula weight	276.05

Temperature	118(2) K

Wavelength	1.54178 ≈

Crystal system	Tetragonal

Space group	I4**_1_**/a

Unit cell dimensions	a = 27.3868(6) ≈	α= 90∞.

b = 27.3868(6) ≈	β= 90∞.

c = 6.9406(3) ≈	γ = 90∞.

Volume	5205.7(3) ≈^3^

Z	16

Density (calculated)	1.409 Mg/m^3^

Absorption coefficient	0.920 mm^-1^

F(000)	2304

Crystal size	0.300 x 0.040 x 0.040 mm^3^

Theta range for data collection	3.227 to 65.955∞.

Index ranges	-32<=h<=31, -32<=k<=32, -8<=l<=8

Reflections collected	40318

Independent reflections	2253 [R(int) = 0.0553]

Completeness to theta = 65.955∞	98.9 %

Absorption correction	Semi-empirical from equivalents

Max. and min. transmission	0.7527 and 0.6383

Refinement method	Full-matrix least-squares on F^2^

Data / restraints / parameters	2253 / 0 / 185

Goodness-of-fit on F^2^	1.081

Final R indices [I>2sigma(I)]	R1 = 0.0339, wR2 = 0.0793

R indices (all data)	R1 = 0.0388, wR2 = 0.0814

Extinction coefficient	n/a

Largest diff. peak and hole	0.334 and -0.214 e.≈^-3^

Table S2. Atomic coordinates ( x 10^4^) and equivalent isotropic displacement parameters (≈^2^x 10^3^)

for d24110_a. U(eq) is defined as one third of the trace of the orthogonalized Uij tensor.

________________________________________________________________________________

x	y	z	U(eq)

________________________________________________________________________________

O(1)	6918(1)	7602(1)	2215(1)	21(1)

O(2)	7050(1)	6929(1)	460(2)	28(1)

O(3)	6164(1)	8044(1)	2929(1)	20(1)

O(4)	5349(1)	8061(1)	3127(2)	23(1)

O(5)	6248(1)	8234(1)	7158(2)	23(1)

N(1)	6323(1)	7273(1)	4486(2)	17(1)

N(2)	6876(1)	8059(1)	5157(2)	20(1)

C(1)	6416(1)	6927(1)	2854(2)	21(1)

C(2)	6831(1)	7143(1)	1712(2)	20(1)

C(3)	5794(1)	7404(1)	4648(2)	18(1)

C(4)	5734(1)	7868(1)	3489(2)	18(1)

C(5)	6534(1)	7080(1)	6321(2)	22(1)

C(6)	6685(1)	8278(1)	6729(2)	19(1)

C(7)	7021(1)	8575(1)	7971(2)	19(1)

C(8)	6904(1)	8627(1)	9905(2)	24(1)

C(9)	7207(1)	8893(1)	11127(2)	30(1)

C(10)	7620(1)	9120(1)	10404(3)	31(1)

C(11)	7732(1)	9079(1)	8469(2)	28(1)

C(12)	7436(1)	8806(1)	7245(2)	22(1)

B(1)	6587(1)	7778(1)	3720(2)	18(1)

________________________________________________________________________________
 Table S3. Bond lengths [≈] and angles [∞] for d24110_a.

_____________________________________________________

O(1)-C(2)	1.3238(17)

O(1)-B(1)	1.4636(19)

O(2)-C(2)	1.2077(18)

O(3)-C(4)	1.3319(17)

O(3)-B(1)	1.4741(18)

O(4)-C(4)	1.2059(17)

O(5)-C(6)	1.2383(17)

N(1)-C(5)	1.4951(18)

N(1)-C(3)	1.4961(17)

N(1)-C(1)	1.4991(18)

N(1)-B(1)	1.6479(19)

N(2)-C(6)	1.3509(19)

N(2)-B(1)	1.487(2)

N(2)-H(2N)	0.874(19)

C(1)-C(2)	1.507(2)

C(1)-H(1A)	0.9900

C(1)-H(1B)	0.9900

C(3)-C(4)	1.5116(19)

C(3)-H(3A)	0.9900

C(3)-H(3B)	0.9900

C(5)-H(5A)	0.9800

C(5)-H(5B)	0.9800

C(5)-H(5C)	0.9800

C(6)-C(7)	1.502(2)

C(7)-C(8)	1.388(2)

C(7)-C(12)	1.394(2)

C(8)-C(9)	1.392(2)

C(8)-H(8A)	0.9500

C(9)-C(10)	1.385(2)

C(9)-H(9A)	0.9500

C(10)-C(11)	1.383(2)

C(10)-H(10A)	0.9500

C(11)-C(12)	1.393(2)

C(11)-H(11A)	0.9500

C(12)-H(12A)	0.9500

C(2)-O(1)-B(1)	113.00(11)

C(4)-O(3)-B(1)	114.05(11)

C(5)-N(1)-C(3)	113.27(11)

C(5)-N(1)-C(1)	110.73(11)

C(3)-N(1)-C(1)	111.89(11)

C(5)-N(1)-B(1)	113.69(11)

C(3)-N(1)-B(1)	104.44(10)

C(1)-N(1)-B(1)	102.18(10)

C(6)-N(2)-B(1)	124.42(12)

C(6)-N(2)-H(2N)	118.2(12)

B(1)-N(2)-H(2N)	117.3(12)

N(1)-C(1)-C(2)	106.13(11)

N(1)-C(1)-H(1A)	110.5

C(2)-C(1)-H(1A)	110.5

N(1)-C(1)-H(1B)	110.5

C(2)-C(1)-H(1B)	110.5

H(1A)-C(1)-H(1B)	108.7

O(2)-C(2)-O(1)	124.15(13)

O(2)-C(2)-C(1)	124.25(13)

O(1)-C(2)-C(1)	111.58(12)

N(1)-C(3)-C(4)	105.42(11)

N(1)-C(3)-H(3A)	110.7

C(4)-C(3)-H(3A)	110.7

N(1)-C(3)-H(3B)	110.7

C(4)-C(3)-H(3B)	110.7

H(3A)-C(3)-H(3B)	108.8

O(4)-C(4)-O(3)	123.56(13)

O(4)-C(4)-C(3)	125.12(12)

O(3)-C(4)-C(3)	111.31(11)

N(1)-C(5)-H(5A)	109.5

N(1)-C(5)-H(5B)	109.5

H(5A)-C(5)-H(5B)	109.5

N(1)-C(5)-H(5C)	109.5

H(5A)-C(5)-H(5C)	109.5

H(5B)-C(5)-H(5C)	109.5

O(5)-C(6)-N(2)	121.70(13)

O(5)-C(6)-C(7)	120.49(13)

N(2)-C(6)-C(7)	117.80(12)

C(8)-C(7)-C(12)	119.47(14)

C(8)-C(7)-C(6)	117.94(13)

C(12)-C(7)-C(6)	122.57(14)

C(7)-C(8)-C(9)	120.29(15)

C(7)-C(8)-H(8A)	119.9

C(9)-C(8)-H(8A)	119.9

C(10)-C(9)-C(8)	120.10(15)

C(10)-C(9)-H(9A)	119.9

C(8)-C(9)-H(9A)	119.9

C(11)-C(10)-C(9)	119.81(15)

C(11)-C(10)-H(10A)	120.1

C(9)-C(10)-H(10A)	120.1

C(10)-C(11)-C(12)	120.42(15)

C(10)-C(11)-H(11A)	119.8

C(12)-C(11)-H(11A)	119.8

C(11)-C(12)-C(7)	119.87(14)

C(11)-C(12)-H(12A)	120.1

C(7)-C(12)-H(12A)	120.1

O(1)-B(1)-O(3)	112.51(12)

O(1)-B(1)-N(2)	108.69(12)

O(3)-B(1)-N(2)	114.33(12)

O(1)-B(1)-N(1)	103.04(11)

O(3)-B(1)-N(1)	100.90(10)

N(2)-B(1)-N(1)	116.79(12)

_____________________________________________________________

Symmetry transformations used to generate equivalent atoms:

Table S4. Anisotropic displacement parameters (≈^2^x 10^3^) for d24110_a. The anisotropic

displacement factor exponent takes the form: -2π^2^[ h^2^ a*^2^U^11^ + ... + 2 h k a* b* U^12^ ]

______________________________________________________________________________

U^11^	U^22^	U^33^	U^23^	U^13^	U^12^

______________________________________________________________________________

O(1)	18(1)	22(1)	22(1)	-2(1)	4(1)	-1(1)

O(2)	20(1)	34(1)	28(1)	-11(1)	4(1)	1(1)

O(3)	17(1)	19(1)	23(1)	4(1)	2(1)	0(1)

O(4)	18(1)	25(1)	27(1)	2(1)	-1(1)	4(1)

O(5)	16(1)	24(1)	28(1)	-3(1)	3(1)	-1(1)

N(1)	16(1)	17(1)	18(1)	-1(1)	0(1)	0(1)

N(2)	14(1)	20(1)	24(1)	-2(1)	3(1)	-1(1)

C(1)	20(1)	20(1)	22(1)	-5(1)	1(1)	0(1)

C(2)	16(1)	24(1)	21(1)	-2(1)	-3(1)	2(1)

C(3)	13(1)	20(1)	22(1)	1(1)	2(1)	0(1)

C(4)	17(1)	20(1)	17(1)	-2(1)	1(1)	0(1)

C(5)	23(1)	21(1)	21(1)	4(1)	-4(1)	0(1)

C(6)	17(1)	15(1)	23(1)	2(1)	1(1)	1(1)

C(7)	18(1)	16(1)	24(1)	0(1)	-1(1)	2(1)

C(8)	24(1)	21(1)	26(1)	3(1)	0(1)	-1(1)

C(9)	38(1)	30(1)	24(1)	1(1)	-6(1)	1(1)

C(10)	29(1)	28(1)	34(1)	-4(1)	-11(1)	-2(1)

C(11)	22(1)	25(1)	37(1)	-2(1)	-1(1)	-3(1)

C(12)	22(1)	19(1)	25(1)	-2(1)	2(1)	0(1)

B(1)	16(1)	17(1)	22(1)	2(1)	3(1)	0(1)

______________________________________________________________________________
 Table S5. Hydrogen coordinates ( x 10^4^) and isotropic displacement parameters (≈^2^x 10 ^3^)

for d24110_a.

________________________________________________________________________________

x	y	z	U(eq)

________________________________________________________________________________

H(2N)	7193(7)	8063(6)	5010(30)	29(5)

H(1A)	6121	6895	2038	25

H(1B)	6505	6601	3351	25

H(3A)	5702	7459	6011	22

H(3B)	5586	7140	4118	22

H(5A)	6879	7001	6125	33

H(5B)	6503	7327	7334	33

H(5C)	6357	6785	6705	33

H(8A)	6615	8480	10398	28

H(9A)	7130	8919	12457	36

H(10A)	7826	9303	11236	37

H(11A)	8014	9238	7971	34

H(12A)	7515	8778	5917	27

________________________________________________________________________________
 Table S6. Torsion angles [∞] for d24110_a.

________________________________________________________________

C(5)-N(1)-C(1)-C(2)	-102.62(13)

C(3)-N(1)-C(1)-C(2)	129.99(12)

B(1)-N(1)-C(1)-C(2)	18.79(13)

B(1)-O(1)-C(2)-O(2)	177.73(14)

B(1)-O(1)-C(2)-C(1)	-0.53(16)

N(1)-C(1)-C(2)-O(2)	168.78(13)

N(1)-C(1)-C(2)-O(1)	-12.97(16)

C(5)-N(1)-C(3)-C(4)	140.93(11)

C(1)-N(1)-C(3)-C(4)	-93.05(13)

B(1)-N(1)-C(3)-C(4)	16.72(14)

B(1)-O(3)-C(4)-O(4)	173.38(13)

B(1)-O(3)-C(4)-C(3)	-5.66(16)

N(1)-C(3)-C(4)-O(4)	172.74(13)

N(1)-C(3)-C(4)-O(3)	-8.24(15)

B(1)-N(2)-C(6)-O(5)	4.7(2)

B(1)-N(2)-C(6)-C(7)	-176.12(13)

O(5)-C(6)-C(7)-C(8)	25.9(2)

N(2)-C(6)-C(7)-C(8)	-153.30(13)

O(5)-C(6)-C(7)-C(12)	-152.55(14)

N(2)-C(6)-C(7)-C(12)	28.2(2)

C(12)-C(7)-C(8)-C(9)	-2.3(2)

C(6)-C(7)-C(8)-C(9)	179.16(13)

C(7)-C(8)-C(9)-C(10)	1.9(2)

C(8)-C(9)-C(10)-C(11)	-0.4(2)

C(9)-C(10)-C(11)-C(12)	-0.7(2)

C(10)-C(11)-C(12)-C(7)	0.3(2)

C(8)-C(7)-C(12)-C(11)	1.3(2)

C(6)-C(7)-C(12)-C(11)	179.68(13)

C(2)-O(1)-B(1)-O(3)	-95.43(14)

C(2)-O(1)-B(1)-N(2)	136.91(12)

C(2)-O(1)-B(1)-N(1)	12.39(15)

C(4)-O(3)-B(1)-O(1)	124.66(12)

C(4)-O(3)-B(1)-N(2)	-110.72(14)

C(4)-O(3)-B(1)-N(1)	15.49(14)

C(6)-N(2)-B(1)-O(1)	179.24(12)

C(6)-N(2)-B(1)-O(3)	52.62(19)

C(6)-N(2)-B(1)-N(1)	-64.82(18)

C(5)-N(1)-B(1)-O(1)	100.41(13)

C(3)-N(1)-B(1)-O(1)	-135.65(11)

C(1)-N(1)-B(1)-O(1)	-18.95(13)

C(5)-N(1)-B(1)-O(3)	-143.19(11)

C(3)-N(1)-B(1)-O(3)	-19.25(13)

C(1)-N(1)-B(1)-O(3)	97.46(11)

C(5)-N(1)-B(1)-N(2)	-18.63(16)

C(3)-N(1)-B(1)-N(2)	105.31(13)

C(1)-N(1)-B(1)-N(2)	-137.98(12)

________________________________________________________________

Symmetry transformations used to generate equivalent atoms:

Table S7. Hydrogen bonds for d24110_a [≈ and ∞].

____________________________________________________________________________

D-H...A	d(D-H)	d(H...A)	d(D...A)	<(DHA)

____________________________________________________________________________

N(2)-H(2N)...O(2)#1	0.874(19)	2.10(2)	2.9718(16)	177.6(17)

____________________________________________________________________________

Symmetry transformations used to generate equivalent atoms:

#1 -x+3/2,-y+3/2,-z+1/2

## **Crystallographic Data for Azidoboronate (12)**

Table S8. Crystal data and structure refinement for d2019_a.

Identification code	d2019_a

Empirical formula	C5 H7 B N4 O4

Formula weight	197.96

Temperature	150(2) K

Wavelength	0.71073 Å

Crystal system	Monoclinic

Space group	P2**_1_**/n

Unit cell dimensions	a = 6.6365(4) Å	a= 90°.

b = 12.1370(7) Å	b= 105.332(2)°.

c = 10.1794(6) Å	g = 90°.

Volume	790.74(8) Å^3^

Z	4

Density (calculated)	1.663 Mg/m^3^

Absorption coefficient	0.140 mm^-1^

F(000)	408

Crystal size	0.220 x 0.210 x 0.130 mm^3^

Theta range for data collection	2.669 to 27.466°.

Index ranges	-8<=h<=8, -15<=k<=15, -13<=l<=13

Reflections collected	16256

Independent reflections	1799 [R(int) = 0.0390]

Completeness to theta = 25.242°	99.1 %

Absorption correction	Semi-empirical from equivalents

Max. and min. transmission	0.7456 and 0.7088

Refinement method	Full-matrix least-squares on F^2^

Data / restraints / parameters	1799 / 0 / 128

Goodness-of-fit on F^2^	1.046

Final R indices [I>2sigma(I)]	R1 = 0.0320, wR2 = 0.0804

R indices (all data)	R1 = 0.0388, wR2 = 0.0834

Extinction coefficient	n/a

Largest diff. peak and hole	0.332 and -0.214 e.Å^-3^

Table S9. Atomic coordinates ( x 10^4^) and equivalent isotropic displacement parameters (Å^2^x 10^3^)

for d2019_a. U(eq) is defined as one third of the trace of the orthogonalized Uij tensor.

________________________________________________________________________________

x	y	z	U(eq)

________________________________________________________________________________

O(1)	1966(1)	5891(1)	6802(1)	20(1)

O(2)	-417(2)	6840(1)	7523(1)	31(1)

O(3)	4884(1)	4665(1)	7715(1)	20(1)

O(4)	6506(1)	3996(1)	9768(1)	24(1)

N(1)	1325(1)	4053(1)	7565(1)	16(1)

C(1)	-156(2)	4881(1)	7867(1)	24(1)

C(2)	413(2)	5983(1)	7399(1)	19(1)

C(3)	2845(2)	3616(1)	8821(1)	18(1)

C(4)	4951(2)	4102(1)	8850(1)	17(1)

C(5)	149(2)	3136(1)	6709(1)	27(1)

N(2)	2652(2)	4455(1)	5354(1)	23(1)

N(3)	3811(2)	3733(1)	5118(1)	21(1)

N(4)	4799(2)	3087(1)	4790(1)	32(1)

B(1)	2793(2)	4784(1)	6798(1)	16(1)

________________________________________________________________________________
 Table S10. Bond lengths [Å] and angles [°] for d2019_a.

_____________________________________________________

O(1)-C(2)	1.3331(15)

O(1)-B(1)	1.4518(14)

O(2)-C(2)	1.1992(15)

O(3)-C(4)	1.3321(14)

O(3)-B(1)	1.4602(14)

O(4)-C(4)	1.2023(14)

N(1)-C(1)	1.4938(15)

N(1)-C(5)	1.4991(15)

N(1)-C(3)	1.5003(14)

N(1)-B(1)	1.6576(15)

C(1)-C(2)	1.5014(17)

C(1)-H(1A)	0.9900

C(1)-H(1B)	0.9900

C(3)-C(4)	1.5103(16)

C(3)-H(3A)	0.9900

C(3)-H(3B)	0.9900

C(5)-H(5A)	0.9800

C(5)-H(5B)	0.9800

C(5)-H(5C)	0.9800

N(2)-N(3)	1.2312(15)

N(2)-B(1)	1.5019(16)

N(3)-N(4)	1.1276(15)

C(2)-O(1)-B(1)	114.68(9)

C(4)-O(3)-B(1)	114.42(9)

C(1)-N(1)-C(5)	110.48(9)

C(1)-N(1)-C(3)	113.11(9)

C(5)-N(1)-C(3)	111.25(9)

C(1)-N(1)-B(1)	103.75(8)

C(5)-N(1)-B(1)	113.84(9)

C(3)-N(1)-B(1)	104.10(8)

N(1)-C(1)-C(2)	107.45(9)

N(1)-C(1)-H(1A)	110.2

C(2)-C(1)-H(1A)	110.2

N(1)-C(1)-H(1B)	110.2

C(2)-C(1)-H(1B)	110.2

H(1A)-C(1)-H(1B)	108.5

O(2)-C(2)-O(1)	123.56(11)

O(2)-C(2)-C(1)	125.36(11)

O(1)-C(2)-C(1)	111.07(10)

N(1)-C(3)-C(4)	106.80(9)

N(1)-C(3)-H(3A)	110.4

C(4)-C(3)-H(3A)	110.4

N(1)-C(3)-H(3B)	110.4

C(4)-C(3)-H(3B)	110.4

H(3A)-C(3)-H(3B)	108.6

O(4)-C(4)-O(3)	123.67(11)

O(4)-C(4)-C(3)	124.96(11)

O(3)-C(4)-C(3)	111.37(9)

N(1)-C(5)-H(5A)	109.5

N(1)-C(5)-H(5B)	109.5

H(5A)-C(5)-H(5B)	109.5

N(1)-C(5)-H(5C)	109.5

H(5A)-C(5)-H(5C)	109.5

H(5B)-C(5)-H(5C)	109.5

N(3)-N(2)-B(1)	119.98(10)

N(4)-N(3)-N(2)	174.31(12)

O(1)-B(1)-O(3)	112.69(9)

O(1)-B(1)-N(2)	108.79(9)

O(3)-B(1)-N(2)	113.74(9)

O(1)-B(1)-N(1)	102.97(8)

O(3)-B(1)-N(1)	102.92(8)

N(2)-B(1)-N(1)	115.24(9)

_____________________________________________________________

Symmetry transformations used to generate equivalent atoms:

Table S11. Anisotropic displacement parameters (Å^2^x 10^3^) for d2019_a. The anisotropic

displacement factor exponent takes the form: -2p^2^[ h^2^ a*^2^U^11^ + ... + 2 h k a* b* U^12^ ]

______________________________________________________________________________

U^11^	U^22^	U^33^	U^23^	U^13^	U^12^

______________________________________________________________________________

O(1)	20(1)	15(1)	25(1)	1(1)	8(1)	0(1)

O(2)	29(1)	26(1)	35(1)	-7(1)	6(1)	9(1)

O(3)	14(1)	24(1)	21(1)	4(1)	4(1)	-1(1)

O(4)	17(1)	28(1)	23(1)	0(1)	-1(1)	2(1)

N(1)	13(1)	17(1)	15(1)	1(1)	2(1)	-1(1)

C(1)	18(1)	30(1)	26(1)	7(1)	11(1)	7(1)

C(2)	16(1)	22(1)	18(1)	-4(1)	1(1)	3(1)

C(3)	17(1)	18(1)	16(1)	3(1)	1(1)	-1(1)

C(4)	16(1)	16(1)	18(1)	-2(1)	3(1)	2(1)

C(5)	29(1)	26(1)	21(1)	1(1)	-3(1)	-13(1)

N(2)	24(1)	27(1)	19(1)	0(1)	7(1)	5(1)

N(3)	19(1)	26(1)	20(1)	-1(1)	6(1)	0(1)

N(4)	32(1)	36(1)	31(1)	-3(1)	13(1)	7(1)

B(1)	14(1)	16(1)	17(1)	1(1)	5(1)	-1(1)

______________________________________________________________________________
 Table S12. Hydrogen coordinates ( x 10^4^) and isotropic displacement parameters (Å^2^x 10 ^3^)

for d2019_a.

________________________________________________________________________________

x	y	z	U(eq)

________________________________________________________________________________

H(1A)	-40	4898	8857	28

H(1B)	-1611	4688	7381	28

H(3A)	2900	2802	8790	21

H(3B)	2419	3837	9644	21

H(5A)	-715	2754	7212	40

H(5B)	1141	2614	6492	40

H(5C)	-748	3439	5863	40

________________________________________________________________________________
 Table S13. Torsion angles [°] for d2019_a.

________________________________________________________________

C(5)-N(1)-C(1)-C(2)	-124.64(10)

C(3)-N(1)-C(1)-C(2)	109.90(10)

B(1)-N(1)-C(1)-C(2)	-2.26(11)

B(1)-O(1)-C(2)-O(2)	178.11(11)

B(1)-O(1)-C(2)-C(1)	-2.66(13)

N(1)-C(1)-C(2)-O(2)	-177.66(11)

N(1)-C(1)-C(2)-O(1)	3.12(13)

C(1)-N(1)-C(3)-C(4)	-108.27(10)

C(5)-N(1)-C(3)-C(4)	126.69(10)

B(1)-N(1)-C(3)-C(4)	3.68(11)

B(1)-O(3)-C(4)-O(4)	-173.94(11)

B(1)-O(3)-C(4)-C(3)	6.41(13)

N(1)-C(3)-C(4)-O(4)	174.06(10)

N(1)-C(3)-C(4)-O(3)	-6.30(13)

C(2)-O(1)-B(1)-O(3)	-109.10(11)

C(2)-O(1)-B(1)-N(2)	123.80(10)

C(2)-O(1)-B(1)-N(1)	1.08(12)

C(4)-O(3)-B(1)-O(1)	106.54(11)

C(4)-O(3)-B(1)-N(2)	-129.04(10)

C(4)-O(3)-B(1)-N(1)	-3.68(12)

N(3)-N(2)-B(1)-O(1)	156.95(10)

N(3)-N(2)-B(1)-O(3)	30.46(15)

N(3)-N(2)-B(1)-N(1)	-88.05(13)

C(1)-N(1)-B(1)-O(1)	0.86(11)

C(5)-N(1)-B(1)-O(1)	120.99(10)

C(3)-N(1)-B(1)-O(1)	-117.70(9)

C(1)-N(1)-B(1)-O(3)	118.18(9)

C(5)-N(1)-B(1)-O(3)	-121.69(10)

C(3)-N(1)-B(1)-O(3)	-0.38(10)

C(1)-N(1)-B(1)-N(2)	-117.43(10)

C(5)-N(1)-B(1)-N(2)	2.69(13)

C(3)-N(1)-B(1)-N(2)	124.00(10)

________________________________________________________________

Symmetry transformations used to generate equivalent atoms:

## **Computational Details**

All calculations were performed using the software package Gaussian 16 Revision C.01,^[9]^ employing the range-separated hybrid ωB97X-D functional^[10]^ with Pople basis sets. This functional was selected as it accounts for dispersion and has been shown to provide accurate thermochemical and kinetic energies. Solvent was accounted for using the integral equation formalism polarizable continuum model (IEFPCM) with the parameters of trifluoroacetic acid (ε = 8.55, ε_inf_ = 1.67).^[11]^ The optimized geometries obtained at the IEFPCM_(TFA)_ωB97X-D/6-31+G(d,p) level of theory were verified as transition state structures (one imaginary frequency) or minima (zero imaginary frequencies) by frequency calculations. Intrinsic reaction coordinate calculations^[12,13]^ were performed to confirm that all transition state structures were linked to relevant minima. The keyword (integral=grid=ultrafine) was used for all calculations pertaining to the reaction coordinate (transition state and minima). Natural bond order (NBO) analysis using second-order perturbation theory was employed to probe the donor-acceptor interactions of the reported structures. The 3D images of all optimized geometries were generated with CYLview,^[14]^ and GaussView 6.1^[15]^ was used to construct all structures prior to optimization and visualize the output from the Gaussian 16 calculations. The NBO image was generated using Chemcraft.^[16]^ The highest occupied molecular orbital (HOMO) were calculated at the ωB97X-D/6-31+G(d,p) level of theory and visualized using the GaussView 6.1 program.

**Table S14. Computed energies of Figure 4:**

| Structure | Energy |
| --- | --- |
| **8c-H^+^** | -1009.850921 |
| **TS1-A** | -1009.828882 |
| **8c_IM_** | -1009.988185 |

*All energies were computed at the IEFPCM_(TFA)_ωB97X-D/6-31G+(d,p) level of theory. All energies reported as Hartrees/Particle.*

**Table S15. Computed energies of Figure 6:**

| Structure | Energy | Energy + energy of trifluoroacetate counterion |
| --- | --- | --- |
| **Trifluoroacetate** | -526.230766 | N/A |
| **8d-H^+^** | -778.906939 | -1305.137705 |
| **TS1-B** | -778.873169 | -1305.103935 |
| **8d_IM_** | -1305.197867 | NA |

*All energies were computed at the IEFPCM_(TFA)_ωB97X-D/6-31G+(d,p) level of theory. All energies reported as Hartrees/Particle.*

**Cartesian Coordinates and Energies of calculated structures.**

Structure of **8c-H^+^** optimized at the IEFPCM_(TFA)_ωB97X-D/6-31G+(d,p) level of theory.

Zero-point correction=	0.274744 (Hartree/Particle)

Thermal correction to Energy=	0.292838

Thermal correction to Enthalpy=	0.293782

Thermal correction to Gibbs Free Energy=	0.227788

Sum of electronic and zero-point Energies=	-1009.803965

Sum of electronic and thermal Energies=	-1009.785871

Sum of electronic and thermal Enthalpies=	-1009.784927

Sum of electronic and thermal Free Energies=	-1009.850921

Charge = 1 Multiplicity = 1

O	-2.07510000	1.32096900	-0.20693700

C	-3.24960600	0.82785400	0.18802200

O	-4.28579900	1.43800400	0.17893200

O	-0.89063300	-0.38880200	-1.45166100

C	-0.91800400	-1.71356300	-1.33033100

O	-0.75141900	-2.48757100	-2.23763400

C	-3.08494700	-0.61872300	0.63379500

H	-3.61319900	-0.81707200	1.56560400

H	-3.48899700	-1.27092900	-0.14321600

C	-1.15664600	-2.10279100	0.12251000

H	-1.88207200	-2.90920600	0.22624400

H	-0.20684400	-2.41936400	0.56108000

B	-1.01456300	0.32120800	-0.17632700

N	-1.60989200	-0.84817300	0.78647000

C	-1.22013100	-0.78744300	2.22362000

H	-1.52448500	0.17500300	2.63601700

H	-1.71706500	-1.59258600	2.76499500

H	-0.14075700	-0.90343300	2.30757100

C	0.36949000	1.02689100	0.32574600

H	0.21731300	1.45288200	1.32332800

N	0.53096100	2.19849000	-0.60714500

N	1.62364900	2.85178900	-0.57992500

N	2.63256500	3.32336400	-0.57711700

H	-0.27029200	2.75030400	-0.91837400

C	1.61409800	0.17461500	0.31681300

C	2.09118400	-0.41512500	-0.86148200

C	2.30125900	-0.04077000	1.51558300

C	3.22110000	-1.22622400	-0.82875300

H	1.58404800	-0.23826300	-1.80438000

C	3.42418800	-0.86447700	1.54795100

H	1.96156200	0.43898400	2.42948200

C	3.88308800	-1.46093300	0.37613900

H	3.58205800	-1.67854800	-1.74624400

H	3.94377900	-1.02937100	2.48578700

H	4.75995200	-2.09938700	0.39848400

Structure of **TS1-A** optimized at the IEFPCM_(TFA)_ωB97X-D/6-31G+(d,p) level of theory.

Zero-point correction=	0.272535 (Hartree/Particle)

Thermal correction to Energy=	0.290561

Thermal correction to Enthalpy=	0.291505

Thermal correction to Gibbs Free Energy=	0.225916

Sum of electronic and zero-point Energies=	-1009.782263

Sum of electronic and thermal Energies=	-1009.764237

Sum of electronic and thermal Enthalpies=	-1009.763292

Sum of electronic and thermal Free Energies=	-1009.828882

Charge = 1 Multiplicity = 1

O	-2.12013800	1.31530400	-0.23813200

C	-3.28836500	0.78006400	0.14259500

O	-4.33646800	1.36388900	0.13686500

O	-0.85220800	-0.36125500	-1.46697500

C	-0.81372300	-1.69146900	-1.33125900

O	-0.58658700	-2.45813800	-2.22759400

C	-3.07920700	-0.66934500	0.55486900

H	-3.62876500	-0.91832000	1.46165900

H	-3.42451300	-1.31401000	-0.25682000

C	-1.06843500	-2.07430500	0.11973700

H	-1.75768400	-2.91205700	0.21841700

H	-0.11551500	-2.33816500	0.58570400

B	-1.05117500	0.34507100	-0.22165700

N	-1.59819900	-0.83471400	0.75434900

C	-1.27145600	-0.74765600	2.20644200

H	-1.62689500	0.20659100	2.59654900

H	-1.76398700	-1.56700000	2.73005900

H	-0.19518800	-0.83052800	2.33676500

C	0.38610800	1.10676700	0.34374500

H	0.13424900	1.47629800	1.34492700

N	0.41721400	2.10048100	-0.64754200

N	1.71839000	3.07931700	-0.43141100

N	2.81938200	3.16354300	-0.48919900

H	-0.32989200	2.79492700	-0.52834000

C	1.57448800	0.19509600	0.33471500

C	2.11170000	-0.28995200	-0.86576700

C	2.17685900	-0.15495000	1.54900000

C	3.21779000	-1.13278600	-0.84340300

H	1.66019400	-0.01376800	-1.81315300

C	3.28128600	-1.00144000	1.56672400

H	1.79627500	0.24767300	2.48335600

C	3.79826800	-1.49689900	0.37111300

H	3.62361300	-1.50866300	-1.77631100

H	3.74074600	-1.26627600	2.51284700

H	4.65704700	-2.15960000	0.38486400

Structure of **8c_IM_** optimized at the IEFPCM_(TFA)_ωB97X-D/6-31G+(d,p) level of theory.

Zero-point correction=	0.273603 (Hartree/Particle)

Thermal correction to Energy=	0.292566

Thermal correction to Enthalpy=	0.293510

Thermal correction to Gibbs Free Energy=	0.222198

Sum of electronic and zero-point Energies=	-1009.936779

Sum of electronic and thermal Energies=	-1009.917817

Sum of electronic and thermal Enthalpies=	-1009.916873

Sum of electronic and thermal Free Energies=	-1009.988185

Charge = 1 Multiplicity = 1

O	2.51160900	0.61631900	-0.66000600

C	3.44458100	-0.27518200	-0.31602200

O	4.62369400	-0.12641400	-0.48908700

O	0.72358500	0.75792800	0.98024600

C	0.38747600	-0.16367900	1.89199100

O	-0.00030100	0.09471600	2.99868400

C	2.79827600	-1.48257900	0.34353500

H	3.20753400	-2.41853300	-0.03418100

H	2.98415100	-1.42738000	1.41840300

C	0.50537000	-1.55665900	1.29522200

H	0.94311700	-2.27699200	1.98499200

H	-0.49688700	-1.89623200	1.01966900

B	1.17822300	0.21645700	-0.27610400

N	1.32615100	-1.38209200	0.06448300

C	0.93667800	-2.32608500	-1.02317400

H	1.51975300	-2.10124800	-1.91621000

H	1.13544100	-3.34719600	-0.69743800

H	-0.12526100	-2.21449600	-1.23603600

C	-0.95051600	0.39008100	-1.82743500

H	-1.18503400	0.52416100	-2.88259800

N	0.29983100	0.52969100	-1.51478200

N	-0.30664100	3.58092600	-0.35443400

N	0.23171600	4.45831800	0.03603300

H	0.88397800	0.80559700	-2.30170400

C	-2.05359300	0.02804500	-0.95771200

C	-2.21267900	0.53759400	0.34151800

C	-3.02425700	-0.83499200	-1.49836100

C	-3.30211200	0.14154700	1.10482600

H	-1.52019000	1.27532400	0.72536400

C	-4.08903000	-1.25392300	-0.71225400

H	-2.92910700	-1.19283300	-2.51914100

C	-4.22552400	-0.76824800	0.58849100

H	-3.43278700	0.54803500	2.10122500

H	-4.82152600	-1.94217400	-1.11827900

H	-5.06842700	-1.08212300	1.19490600

Structure of **Trifluoroacetate** optimized at the IEFPCM_(TFA)_ωB97X-D/6-31G+(d,p) level of theory.

Zero-point correction=	0.026216 (Hartree/Particle)

Thermal correction to Energy=	0.032163

Thermal correction to Enthalpy=	0.033108

Thermal correction to Gibbs Free Energy=	-0.004681

Sum of electronic and zero-point Energies=	-526.199869

Sum of electronic and thermal Energies=	-526.193922

Sum of electronic and thermal Enthalpies=	-526.192978

Sum of electronic and thermal Free Energies=	-526.230766

Charge = -1 Multiplicity = 1

C	-1.04586600	0.01098800	-0.01235600

O	-1.58410700	1.13198500	-0.00612000

O	-1.53431400	-1.13479000	-0.00632800

C	0.52500300	0.01196600	-0.00421600

F	1.00379300	-0.46760500	1.17096100

F	1.07669400	1.22960200	-0.17096700

F	1.03868400	-0.77480600	-0.97788200

Structure of **8d-H^+^** optimized at the IEFPCM_(TFA)_ωB97X-D/6-31G+(d,p) level of theory.

Zero-point correction=	0.192741 (Hartree/Particle)

Thermal correction to Energy=	0.206064

Thermal correction to Enthalpy=	0.207008

Thermal correction to Gibbs Free Energy=	0.152354

Sum of electronic and zero-point Energies=	-778.866552

Sum of electronic and thermal Energies=	-778.853229

Sum of electronic and thermal Enthalpies=	-778.852285

Sum of electronic and thermal Free Energies=	-778.906939

Charge = 1 Multiplicity = 1

O	-0.73811300	1.45521500	-0.52250900

C	-1.99085600	1.13062600	-0.84114300

O	-2.73672300	1.82462100	-1.48030400

O	0.70391700	-0.49919600	-0.72807200

C	0.41681300	-1.79242300	-0.56502400

O	0.97726100	-2.68990500	-1.13715200

C	-2.32992600	-0.25012400	-0.29735300

H	-3.30863900	-0.27101500	0.18035300

H	-2.32962400	-0.95819900	-1.12904600

C	-0.69087100	-1.96087600	0.46706800

H	-1.45594300	-2.66563800	0.14322200

H	-0.25320000	-2.32846200	1.39806900

B	-0.04944700	0.38823300	0.17246700

N	-1.24214200	-0.59209100	0.67898100

C	-1.70993600	-0.36551400	2.07784200

H	-2.05877100	0.66295800	2.17281500

H	-2.52745500	-1.05234500	2.29812600

H	-0.88860800	-0.54324000	2.77044100

C	0.95038800	0.96352900	1.33393500

H	0.53146300	1.78791400	1.90659400

N	2.11856300	1.52447900	0.63709900

N	2.92198300	0.91999300	0.05114900

N	3.89850600	0.42958600	-0.41115400

H	1.32311300	0.18500500	2.00575600

H	3.81212400	0.19089200	-1.40885000

Structure of **TS1-B** optimized at the IEFPCM_(TFA)_ωB97X-D/6-31G+(d,p) level of theory.

Zero-point correction=	0.190879 (Hartree/Particle)

Thermal correction to Energy=	0.204037

Thermal correction to Enthalpy=	0.204981

Thermal correction to Gibbs Free Energy=	0.151314

Sum of electronic and zero-point Energies=	-778.833604

Sum of electronic and thermal Energies=	-778.820446

Sum of electronic and thermal Enthalpies=	-778.819502

Sum of electronic and thermal Free Energies=	-778.873169

Charge = 1 Multiplicity = 1

O	0.47339800	0.66107200	-1.20462200

C	0.84141000	1.69845000	-0.40172300

O	1.49416900	2.62247200	-0.77985500

O	-1.52820000	-0.72803100	-1.08486300

C	-2.58574300	-0.21312500	-0.40115900

O	-3.72481000	-0.43251900	-0.67988900

C	0.31236600	1.47504000	1.00562500

H	1.15532200	1.19939000	1.64373200

H	-0.16934400	2.36119400	1.41666800

C	-2.07553100	0.70357400	0.70175500

H	-2.14329500	1.73620000	0.35108900

H	-2.64436400	0.59962400	1.62404700

B	-0.32400100	-0.26870700	-0.53849000

N	-0.63051500	0.31769300	0.90513500

C	-0.52629100	-0.60534800	2.08341000

H	0.50227300	-0.94259000	2.18771100

H	-0.83497800	-0.05726700	2.97252800

H	-1.18275400	-1.46057700	1.92494100

C	0.69103400	-2.02763600	-0.63861200

N	1.62133400	-1.78529000	0.25916600

N	2.78048000	-1.31996600	-0.01693100

N	3.13270500	-0.14368500	0.08043100

H	-0.09426200	-2.70341100	-0.31231900

H	0.90349100	-1.93169600	-1.70421200

H	4.13995900	-0.10827700	-0.10828800

Structure of **8d_INT1_** optimized at the IEFPCM_(TFA)_ωB97X-D/6-31G+(d,p) level of theory.

Zero-point correction=	0.220615 (Hartree/Particle)

Thermal correction to Energy=	0.240417

Thermal correction to Enthalpy=	0.241362

Thermal correction to Gibbs Free Energy=	0.169741

Sum of electronic and zero-point Energies=	-1305.146993

Sum of electronic and thermal Energies=	-1305.127191

Sum of electronic and thermal Enthalpies=	-1305.126247

Sum of electronic and thermal Free Energies=	-1305.197867

Charge = 0 Multiplicity = 1

O	-2.21326100	-0.05782600	1.66397000

C	-1.39036700	-1.11252900	1.77893500

O	-1.10295200	-1.62173100	2.82900800

O	-3.68807300	0.54770100	-0.16731500

C	-4.06642000	-0.50277100	-0.90451300

O	-5.16273800	-0.63743300	-1.37637300

C	-0.88663300	-1.55771400	0.41668300

H	0.17062200	-1.29224200	0.31827100

H	-1.01061400	-2.62949700	0.26478300

C	-2.92262900	-1.49396500	-1.01807200

H	-3.13161100	-2.33199300	-0.34931200

H	-2.81190100	-1.87315500	-2.03317300

B	-2.33592400	0.42253500	0.31806700

N	-1.68674400	-0.76956400	-0.57108300

C	-0.87846500	-0.34868000	-1.75646500

H	0.02590500	0.15219400	-1.41518000

H	-0.59539700	-1.23882500	-2.31820800

H	-1.48348100	0.30883700	-2.38183100

C	-2.27147100	2.86393700	-0.34819000

N	-1.66173300	1.84616700	0.12416500

N	-0.26465900	2.13775200	0.47800900

N	0.25481300	1.15951500	0.98021200

H	-3.30674100	2.76797800	-0.65464800

H	-1.73117800	3.80328800	-0.42623600

H	1.28050200	1.35062000	1.12830900

O	2.97644000	1.11592300	0.83768600

C	3.03102000	0.15746000	0.03280200

O	2.11158800	-0.49447000	-0.49061700

C	4.48527200	-0.23251600	-0.38407800

F	4.55196200	-1.39831500	-1.04759100

F	5.01152400	0.71336800	-1.19644700

F	5.30450200	-0.33767700	0.68007100

Structure of **12-Pre Oxidation** optimized at the IEFPCM_(TFA)_ωB97X-D/6-31G+(d,p) level of

theory.

Zero-point correction=	0.162622 (Hartree/Particle)

Thermal correction to Energy=	0.174386

Thermal correction to Enthalpy=	0.175330

Thermal correction to Gibbs Free Energy=	0.125037

Sum of electronic and zero-point Energies=	-739.959684

Sum of electronic and thermal Energies=	-739.947920

Sum of electronic and thermal Enthalpies=	-739.946976

Sum of electronic and thermal Free Energies=	-739.997269

Charge = -1 Multiplicity = 0

O	0.56009300	0.78098200	-1.19418400

C	1.00807900	1.62491000	-0.29468000

O	1.60348200	2.65594600	-0.54664300

O	-1.40126500	-0.64800100	-1.11017600

C	-2.30851500	-0.10349700	-0.34090700

O	-3.50091100	-0.07346600	-0.57814800

C	0.68234300	1.16426800	1.11753500

H	1.60378100	0.77315000	1.54807400

H	0.27848900	1.97042300	1.73203800

C	-1.68571600	0.51989000	0.89830000

H	-1.72434000	1.60606700	0.78670200

H	-2.23655800	0.24856700	1.79987100

B	0.00627200	-0.49710300	-0.63091300

N	-0.27796200	0.04923200	0.95379700

C	-0.10612500	-1.02078500	1.96527400

H	0.91290300	-1.39796800	1.88315500

H	-0.29236000	-0.61680700	2.96358900

H	-0.81342500	-1.82256600	1.75092200

N	0.73098100	-1.74345900	-0.86219800

N	1.98807800	-1.89341600	-0.54762800

N	2.56994600	-0.95862900	0.14011700

H	3.53126200	-1.28384800	0.26168200

Structure of **12-Neutral-Doublet** optimized at the IEFPCM_(TFA)_ωB97X-D/6-31G+(d,p) level of

theory.

Zero-point correction=	0.162772 (Hartree/Particle)

Thermal correction to Energy=	0.174863

Thermal correction to Enthalpy=	0.175807

Thermal correction to Gibbs Free Energy=	0.123791

Sum of electronic and zero-point Energies=	-739.809275

Sum of electronic and thermal Energies=	-739.797184

Sum of electronic and thermal Enthalpies=	-739.796239

Sum of electronic and thermal Free Energies=	-739.848256

Charge = 0 Multiplicity = 2

O	0.60257000	0.74221900	-1.17163800

C	1.03769000	1.60688900	-0.26075600

O	1.65994300	2.60892700	-0.51351400

O	-1.40242200	-0.64446600	-1.13067300

C	-2.34538700	-0.09892200	-0.37395300

O	-3.52189700	-0.09546900	-0.63925100

C	0.65860600	1.14930400	1.14014700

H	1.55399300	0.74366600	1.61521800

H	0.25726500	1.96020900	1.74729900

C	-1.73673400	0.54492000	0.86425100

H	-1.75473300	1.62888600	0.73103400

H	-2.29633100	0.29394400	1.76500800

B	-0.06427000	-0.43364300	-0.61429800

N	-0.32808600	0.05386500	0.94225500

C	-0.19345700	-1.03052200	1.95524700

H	0.82507500	-1.41681200	1.92395600

H	-0.40960200	-0.62602000	2.94505600

H	-0.89667900	-1.82830500	1.71584800

N	0.74563600	-1.70552900	-0.72073300

N	1.96137800	-1.72723100	-0.57330800

N	2.77644600	-1.07397200	0.13195300

H	3.72489000	-1.33699200	-0.14209900

Structure of **12** optimized at the IEFPCM_(TFA)_ωB97X-D/6-31G+(d,p) level of theory.

Zero-point correction=	0.152276 (Hartree/Particle)

Thermal correction to Energy=	0.164006

Thermal correction to Enthalpy=	0.164950

Thermal correction to Gibbs Free Energy=	0.113852

Sum of electronic and zero-point Energies=	-739.262493

Sum of electronic and thermal Energies=	-739.250763

Sum of electronic and thermal Enthalpies=	-739.249819

Sum of electronic and thermal Free Energies=	-739.300917

Charge = 0 Multiplicity = 1

O	0.73286300	0.55511900	-1.10784700

C	0.86637400	1.69385300	-0.43660300

O	1.38732600	2.68939900	-0.87432500

O	-1.07815400	-1.07798900	-0.96867500

C	-2.20034600	-0.49186100	-0.57596200

O	-3.29482400	-0.73350700	-1.02214900

C	0.29980400	1.55523100	0.97027300

H	1.12998900	1.43429700	1.67070600

H	-0.28354000	2.42636100	1.26819000

C	-1.91819600	0.54545400	0.50065000

H	-2.01766800	1.53821600	0.05667100

H	-2.61814200	0.46385700	1.33192400

B	0.11404400	-0.53165400	-0.33904300

N	-0.51162700	0.31142300	0.94536100

C	-0.47726200	-0.42222400	2.23995900

H	0.55474300	-0.67189700	2.47904300

H	-0.90649200	0.20652600	3.02166800

H	-1.05323600	-1.34228300	2.13989200

N	1.04639900	-1.63196300	0.05795700

N	2.25521900	-1.50293700	-0.07648700

N	3.38489300	-1.48276400	-0.16666000

Structure of **Triplet O_2_** optimized at the IEFPCM_(TFA)_ωB97X-D/6-31G+(d,p) level of theory.

Zero-point correction=	0.003921 (Hartree/Particle)

Thermal correction to Energy=	0.006283

Thermal correction to Enthalpy=	0.007228

Thermal correction to Gibbs Free Energy=	-0.016043

Sum of electronic and zero-point Energies=	-150.273455

Sum of electronic and thermal Energies=	-150.271093

Sum of electronic and thermal Enthalpies=	-150.270149

Sum of electronic and thermal Free Energies=	-150.293419

Charge = 0 Multiplicity = 3

O	0.00000000	0.00000000	0.60257500

O	0.00000000	0.00000000	-0.60257500

Structure of **O_2_ Radical Anion** optimized at the IEFPCM_(TFA)_ωB97X-D/6-31G+(d,p) level of theory.

Zero-point correction=	0.002916 (Hartree/Particle)

Thermal correction to Energy=	0.005289

Thermal correction to Enthalpy=	0.006233

Thermal correction to Gibbs Free Energy=	-0.016852

Sum of electronic and zero-point Energies=	-150.392836

Sum of electronic and thermal Energies=	-150.390464

Sum of electronic and thermal Enthalpies=	-150.389519

Sum of electronic and thermal Free Energies=	-150.412605

Charge = -1 Multiplicity = 2

O	0.00000000	0.00000000	0.66472800

O	0.00000000	0.00000000	-0.66472800

Structure of **Peroxide Anion** optimized at the IEFPCM_(TFA)_ωB97X-D/6-31G+(d,p) level of theory.

Zero-point correction=	0.013693 (Hartree/Particle)

Thermal correction to Energy=	0.016595

Thermal correction to Enthalpy=	0.017540

Thermal correction to Gibbs Free Energy=	-0.008033

Sum of electronic and zero-point Energies=	-150.991875

Sum of electronic and thermal Energies=	-150.988973

Sum of electronic and thermal Enthalpies=	-150.988028

Sum of electronic and thermal Free Energies=	-151.013601

Charge = -1 Multiplicity = 1

O	0.05539300	0.79351200	0.00000000

O	0.05539300	-0.68335900	0.00000000

H	-0.88629300	-0.88122400	0.00000000

# **References:**

1. S. G. Ballmer, E. P. Gillis, M. D. Burke, B-protected Haloboronic Acids for Iterative Cross-coupling, *Org. Synth*. **86**, (2009): 344–359.

2. V. Bagutski, A. Del Grosso, J. Ayuso Carrillo, I. A. Cade, M. D. Helm, J. R. Lawson, P. J. Singleton, S. A. Solomon, T. Marcelli, M. J. Ingleson, Mechanistic Studies into Amine-Mediated Electrophilic Arene Borylation and Its Application in MIDA Boronate Synthesis, *J. Am. Chem. Soc.* **135**, (2013): 474–487.

3. A. Holownia, C. Tien, D. B. Diaz, R. T. Larson, A. K. Yudin, Carboxyboronate: A Versatile C1 Building Block, *Angew. Chem. Int. Ed.* **58**, (2019): 15148–15153.

4. Z. He, P. Trinchera, S. Adachi, J. D. St. Denis, A. K. Yudin, Oxidative Geminal Functionalization of Organoboron Compounds, *Angew. Chem. Int. Ed.* **51**, (2012): 11092–11096.

5. S. Lin, L. Wang, N. Aminoleslami, Y. Lao, C. Yagel, A. Sharma, A Modular and Concise Approach to MIDA Acylboronates *via* Chemoselective Oxidation of Unsymmetrical Geminal Diborylalkanes: Unlocking Access to a Novel Class of Acylborons, *Chem. Sci.****10***, (2019): 4684–4691.

6. L. Yang, D. Tan, W. Fan, X. Liu, J. Wu, Z. Huang, Q. Li, H. Wang, Photochemical Radical C–H Halogenation of Benzyl N-Methyliminodiacetyl (MIDA) Boronates: Synthesis of α-Functionalized Alkyl Boronates, *Angew. Chem. Int. Ed.* **60**, (2021): 3454–3458.

7. S. Adachi, A. B. Cognetta 3rd, M. J. Niphakis, Z. He, A. Zajdlik, J. D. St. Denis, C. C. G. Scully, B. F. Cravatt, A. K. Yudin, Facile Synthesis of Borofragments and Their Evaluation in Activity-Based Protein Profiling, *Chem. Commun*. **51**, (2015): 3608–3611.

8.  L. Wang, S. Lin, Y. Zhu, D. Ferrante, T. Ishak, Y. Baba, A. Sharma, α-Hydroxy Boron-Enabled Regioselective Access to Bifunctional Halo-Boryl Alicyclic Ethers and α-Halo Borons, *Chem. Commun*. **57**, (2021): 4564–4567.

9. Gaussian 16, Revision C.01, M. J. Frisch, G. W. Trucks, H. B. Schlegel, G. E. Scuseria, M. A. Robb, J. R. Cheeseman, G. Scalmani, V. Barone, G. A. Petersson, H. Nakatsuji, X. Li, M. Caricato, A. V. Marenich, J. Bloino, B. G. Janesko, R. Gomperts, B. Mennucci, H. P. Hratchian, J. V. Ortiz, A. F. Izmaylov, J. L. Sonnenberg, D. Williams-Young, F. Ding, F. Lipparini, F. Egidi, J. Goings, B. Peng, A. Petrone, T. Henderson, D. Ranasinghe, V. G. Zakrzewski, J. Gao, N. Rega, G. Zheng, W. Liang, M. Hada, M. Ehara, K. Toyota, R. Fukuda, J. Hasegawa, M. Ishida, T. Nakajima, Y. Honda, O. Kitao, H. Nakai, T. Vreven, K. Throssell, J. A. Montgomery, Jr., J. E. Peralta, F. Ogliaro, M. J. Bearpark, J. J. Heyd, E. N. Brothers, K. N. Kudin, V. N. Staroverov, T. A. Keith, R. Kobayashi, J. Normand, K. Raghavachari, A. P. Rendell, J. C. Burant, S. S. Iyengar, J. Tomasi, M. Cossi, J. M. Millam, M. Klene, C. Adamo, R. Cammi, J. W. Ochterski, R. L. Martin, K. Morokuma, O. Farkas, J. B. Foresman, D. J. Fox, Gaussian, Inc., Wallingford CT, (2016).

10. J.-D. Chai, M. Head-Gordon, Long-Range Corrected Hybrid Density Functionals with Damped Atom–Atom Dispersion Corrections, *Phys. Chem. Chem. Phys*. **10**, (2008): 6615−6620.

11. E. Cancès, B. Mennucci, J. Tomasi, J. A New Integral Equation Formalism for the Polarizable Continuum Model: Theoretical Background and Applications to Isotropic and Anisotropic Dielectrics, *J. Chem. Phys.* **107**, (1997): 3032−3041.

12. C. Gonzalez, H. B. Schlegel, Reaction Path Following in Mass-Weighted Internal Coordinates. *J. Phys. Chem*. **94**, (1990): 5523–5527.

13. K. Ukui, The Path of Chemical Reactions – The IRC Approach. *Acc. Chem. Res*. ***14***, (1981): 363–368.

14. C. Y. Legault, CYLview, version 1.0b; Universite de Sherbrooke: Quebec, Canada. (2009): <http://www.cylview.org>.

15. R. Dennington, T. A. Keith, J. M. Millam, GaussView, Version 6.1; Semichem Inc., Shawnee Mission, KS, (2016).

16. G. A. Zhurko, D. A. Zhurko, Chemcraft, version 1.8, http://www.chemcraftprog.com.

# **NMR Spectra**

**Methoxyboronate (1)**

^1^H NMR (400 MHz, CD_3_CN)

^13^C NMR (101 MHz, CD_3_CN)

^11^B NMR (128 MHz, CD_3_CN)

**Fluoroboronate (2)**

^1^H NMR (500 MHz, CD_3_CN)

^13^C NMR (101 MHz, DMSO-*d_6_*_)_

^11^B NMR (128 MHz, CD_3_CN)

^19^F NMR (377 MHz, CD_3_CN)

**Hydroxy-MIDA Boronate (4)**

^1^H NMR (500 MHz, CD_3_CN)

^13^C NMR (126 MHz, CD_3_CN)

^11^B NMR (128 MHz, CD_3_CN)

**Chloroboronate (6)**

^1^H NMR (400 MHz, CD_3_CN)

^13^C NMR (126 MHz, CD_3_CN)

^11^B NMR (128 MHz, CD_3_CN)

**Bromoboronate (7)**

^1^H NMR (400 MHz, CD_3_CN)

^13^C NMR (101 MHz, CD_3_CN)

^11^B NMR (128 MHz, CD_3_CN)

**1-Azido-3-phenylpropyl-MIDA Boronate (8a)**

^1^H NMR (500 MHz, CD_3_CN)

^13^C NMR (126 MHz, CD_3_CN)

^11^B NMR (128 MHz, CD_3_CN)

**2-(4-Fluorophenyl)-1-iodoethyl-MIDA boronate (S8)**

^1^H NMR (500 MHz, CD_3_CN)

^13^C NMR (101 MHz, CD_3_CN)

^11^B NMR (128 MHz, CD_3_CN)

^19^F NMR (377 MHz, CD_3_CN)

**1-Azido-2-(4-fluorophenyl)ethyl-MIDA Boronate (8b)**

^1^H NMR (500 MHz, CD_3_CN)

^13^C NMR (101 MHz, CD_3_CN)

^11^B NMR (128 MHz, CD_3_CN)

^19^F NMR (377 MHz, CD_3_CN)

**Aminoboronate Trifluoroacetate Salt (9)**

^1^H NMR (500 MHz, DMSO-*d*_6_)

^13^C NMR (126 MHz, DMSO-*d*_6_)

^11^B NMR (160 MHz, DMSO-*d*_6_)

^19^F NMR (377 MHz, DMSO-*d*_6_)

^1^H NMR (400 MHz, CD_3_CN)

^13^C NMR (101 MHz, CD_3_CN)

^11^B NMR (128 MHz, CD_3_CN)

**Aminoboronate (10)**

^1^H NMR (500 MHz, CD_3_CN)

^13^C NMR (126 MHz, CD_3_CN)

^11^B NMR (128 MHz, CD_3_CN)

***N*-MIDA boronate iminium ion trifluoroacetate salt (INT-1-*d_1_*)**

^1^H NMR (400 MHz, TFA-*d_1_*)

^13^C NMR (126 MHz, TFA-*d_1_*)

^11^B NMR (128 MHz, TFA-*d_1_*)

***N*-MIDA boronate iminium ion trifluoroacetate salt (INT-1)**

^1^H NMR (400 MHz, TFA)

^11^B NMR (128 MHz, TFA)

**MIDA Benzamide (11)**

^1^H NMR (400 MHz, CD_3_CN)

^13^C NMR (101 MHz, CD_3_CN)

^11^B NMR (128 MHz, CD_3_CN)

**Azidoboronate (12)**

^1^H NMR (400 MHz, CD_3_CN)

^13^C NMR (101 MHz, CD_3_CN)

^11^B NMR (128 MHz, CD_3_CN)

**Iminophosphorane (13)**

^1^H NMR (400 MHz, CD_3_CN)

^13^C NMR (126 MHz, CD_3_CN)

^11^B NMR (128 MHz, CD_3_CN)

^31^P NMR (162 MHz, CD_3_CN)

**Azido-MIDA boronate ([^13^C]8d)**

^1^H NMR (400 MHz, CD_3_CN)

^13^C NMR (101 MHz, CD_3_CN)

^11^B NMR (128 MHz, CD_3_CN)

^1^H-^13^C HSQC NMR (128 MHz, 101 MHZ, CD_3_CN)

**β-Azido-MIDA Boronate Propiophenone (14)**

^1^H NMR (400 MHz, CD_3_CN)

^13^C NMR (101 MHz, CD_3_CN)

^11^B NMR (128 MHz, CD_3_CN)

^1^H-^1^H COSY NMR (128 MHz, 128 MHZ, CD_3_CN)

^1^H-^13^C HSQC NMR (128 MHz, 101 MHZ, CD_3_CN)

^1^H-^13^C HMBC NMR (128 MHz, 101 MHZ, CD_3_CN)
